# The impacts of anthropogenic linear features on the space-use patterns of two sympatric ungulates

**DOI:** 10.1186/s40462-026-00628-y

**Published:** 2026-02-18

**Authors:** Ronan B. Hart, Simona Picardi, Tal Avgar

**Affiliations:** 1 Department of Wildland Resources, Utah State University, Logan, UT USA; 2 Present Address: Department of Biology, University of New Mexico, Albuquerque, NM USA; 3 Present Address: USDA Forest Service, Rocky Mountain Research Station, Albuquerque, NM USA; 4 Present Address: Department of Fish and Wildlife Sciences, University of Idaho, Moscow, ID USA; 5 Present Address: Department of Biology, University of British Columbia, Syilx Okanagan Nation Territory, British Columbia, Canada

**Keywords:** Fence, Habitat availability, Habitat selection, Home range, Home-range size, Home-range compactness, Mule deer, Occurrence distribution, Pronghorn, Resource selection, Road ecology

## Abstract

As human activity increases worldwide, many ecologists have focused on how anthropogenic linear features (ALFs) such as roads and fences impact and disrupt animal space-use behavior and how this disruption could potentially affect population viability. The properties of an animal’s occurrence distribution (OD), namely its size, shape, and habitat associations, reflect the animal’s balance of costs and benefits and thus can act as indirect indicators of behavioral optimality. Measuring deviations from theoretical space-use optimality can provide insight into the non-lethal effects of ALFs on wildlife in different environmental contexts. Here, we focused on the seasonal space-use patterns of two wide-ranging, highly mobile species of great cultural and economic value: mule deer (*Odocoileus hemionus*; *n* = 3105) and pronghorn (*Antilocapra americana*; *n* = 320). We calculated the average use of six environmental and three ALF attributes, weighted by their intensity of use within the OD, and contrasted those with their respective average availability within a 100-km^2^ reference area centered on each animal’s OD. We show that mule deer space-use is more impacted by roads, while pronghorn space-use is affected more by fences, specifically in the winter when snow depth may hinder their ability to cross fences. Our results highlight the dynamic nature of the availability domain and the importance of properly accounting for this dynamism in habitat selection analyses. This research expands on the theoretical literature of animal space use and their response to ALFs in a rapidly changing world and further provides practical strategies for wildlife managers to take when mitigating ALF impacts on their target species.

**Supplementary information:**

The online version contains supplementary material available at 10.1186/s40462-026-00628-y.

## Introduction

As human populations increase worldwide, there are growing efforts to measure the impacts of anthropogenic activity on wildlife. In particular, anthropogenic linear features (ALFs; e.g. roads or fences) are constructed at high densities in regions with otherwise sparse human footprints [1, 2]. In these regions, ALFs may be the only anthropogenic feature many animals encounter regularly, making it critical to understand how ALFs affect individual space use and, consequently, their fitness. While some animals are attracted to ALFs, such as roads, because they are associated with specific resources [3] or because they allow for efficient movement over long distances [4, 5], there is consensus in the literature that ALFs have negative effects on many species of wildlife [6–8]. Besides lethal effects associated with, for example, vehicle collisions on roads [1], predation on seismic lines [4], or entanglement in fences [2, 9], ALFs can also exert non-lethal fitness costs on wildlife by hampering an animal’s ability to access resources [2, 9–13] and avoid risk [4, 14–17]. Measuring such non-lethal effects of ALFs, however, is challenging because fitness costs accumulate through time and are difficult to disentangle from other co-occurring causal factors.

Home ranges can be used as proxies for quantifying non-lethal effects of ALFs on wildlife fitness. The home range is the area an animal uses during its normal, daily activities [18] and manifests from the animal balancing risks and rewards while moving in space [19, 20]. Theory establishes a direct link between space-use decisions, home range properties (relative size and shape in geographic space and composition in environmental space), and an individual’s fitness [21–23]; thus, there is strong theoretical support for the use of home range properties as indirect indicators of behavioral optimality and, thus, fitness. Deviations from theoretical optimality in home range properties should correspond to a proportional decrease in fitness and can provide insight into the non-lethal effects of ALFs on wildlife.

Home-range size is a property emerging from balancing the energy required for an animal’s survival and available energy on the landscape [24]. Theoretically, and all else being equal, smaller home ranges arise in habitats with a high resource quantity and quality compared to habitats without [19, 25–28]. If animals behave optimally, home ranges should be as small as possible to encompass sufficient resources for survival, growth, and reproduction while minimizing the costs associated with obtaining these resources and the risks associated with predation [19]. Factors that affect an animal’s ability to move through the landscape and encounter resources can force animals away from optimal space-use patterns and result in increased home-range size. Thus, when ALFs decrease habitat quality, animals may be forced to move more and farther to acquire the resources they need, and home-range size increases as a result. ALFs that are semi-permeable may also increase home-range size, as animals would be devoting time and energy attempting to cross the barrier, meaning they need to spend more time and move farther to acquire resources to make up for the energy spent [25]. However, when ALFs fully block movement, they constrain animals within smaller areas and may, conversely, decrease home-range size. Thus, the effects of ALFs on home-range size are context-dependent, and positive or negative relationships between ALF density and home-range size do not automatically indicate positive or negative effects of ALFs; inferring on ALF effects based on home range properties requires multiple lines of evidence.

Home-range shapes are determined by the distribution of resources across the landscape and the balance of risk and reward. Theory predicts that home ranges would be completely circular and compact in a landscape with a homogeneous distribution of resources [29]. As animals use space within their home range non-randomly, certain parts of the home range are used at a higher intensity than others [30, 31]; a compact home range helps minimize movement costs between high-intensity use areas. Thus, a compact home range indicates a high intake of resources and a low expenditure of energy to access these resources. When the landscape becomes more heterogeneous or more difficult to move through, home-range shapes are expected to expand, elongate, and become less compact in response [29]. Partially or fully impermeable ALFs may force animals to move in an elongated and complex pattern and deviate from the optimal circular home-range shape, which increases movement costs and leads to less efficient access to core areas.

So far we have focused on a home range’s geometry in geographical space (size and shape), but a home range can also be characterized by its environmental composition (i.e., its ‘shape’ in environmental space), which reflects an animal’s habitat selection. Habitat selection is a functional response whereby the strength of selection for (or avoidance of) conditions, resources, and risks depends on their availability on the landscape [32–37]. Generally, animals are expected to avoid ALFs because ALFs fragment habitat and can pose direct mortality risks [1, 8, 11]. However, as ALF density increases, ALF avoidance is expected to decline because animals are unable to avoid features that are pervasive on the landscape [38]. The pervasiveness of ALFs on the landscape likely also impacts how animals select for other environmental characteristics [39]. For instance, according to the risk-disturbance hypothesis, animals may perceive anthropogenic disturbances as risky and react similarly as they would to an actual predator [15]; because animals balance trade-offs of risks and reward, an abundance of risks could compromise an animal’s normal habitat selection behaviors.

**Fig. 1:**
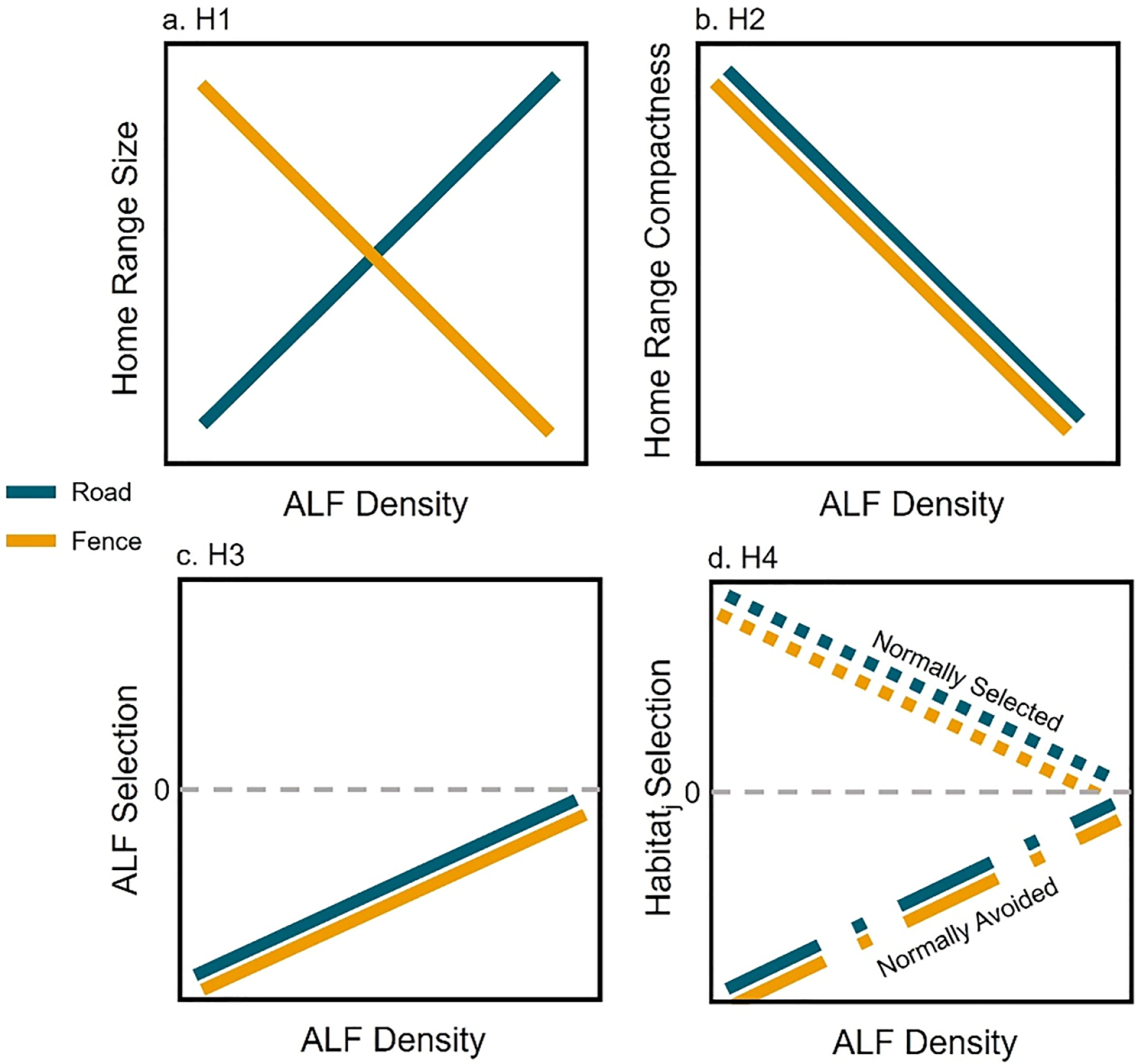
Conceptual figures of predicted responses of (**a**) home-range size, (**b**) home-range shape (compactness), (**c**) selection of anthropogenic linear features (ALFs), and (**d**) selection or avoidance of other habitat attributes (dotted line representing attributes that are normally selected for and dashed-dot line representing attributes that are normally avoided) as a function of ALF density

Building on the existing theory, this study aims to understand how the home range characteristics (in both geographical and environmental spaces) of two sympatric ungulate species change in response to ALF density–specifically roads and fences. We generated hypotheses based on previous literature on ALF effects on wildlife, and we developed predictions based on theoretical expectations of how deviations from behavioral optimality should manifest through home range characteristics. While ALFs have been shown to affect wildlife across taxa, responses to ALFs are taxon- or even species-specific [7]; thus, we developed our hypotheses based on the literature on ungulates. We tested four hypotheses: (H1) roads are permeable barriers that reduce access to resources by fragmenting and degrading habitat [10, 40]. Therefore, we predicted that roads force ungulates to move more and further in search of foraging opportunities, and thus home range size should increase in response to increased road density. In contrast, because fences are less permeable and more likely to block movement [13, 41], we predicted home-range size to decrease in response to increasing fence density (Fig. 1a). (H2) Due to the semi-permeable to permeable nature of ALFs, we predicted home ranges to become more complex, elongated, and less compact as ALF density increases (Fig. 1b). (H3) Many animals, and most ungulates, avoid ALFs [10, 11, 13, 42], but may become habituated as they become more common (the ‘relaxed avoidance’ hypothesis, sensu [34, 43]). We predicted that ungulates would avoid ALFs at a low ALF density, but as ALF density increases on the landscape, avoidance behavior would erode because ALFs become so pervasive that they are impossible to avoid (Fig. 1c). (H4) As ALF density increases, the animal’s selection for or avoidance of other habitat characteristics should become compromised. We predicted that selection strength for habitat attributes would approach zero (i.e. no selection nor avoidance) in response to increasing ALF density (Fig. 1d), marking a deviation from natural behavioral patterns. Through the combination of these four hypotheses (multiple lines of evidence), rather than each hypothesis on its own, we will be able to draw conclusions of the impacts of ALFs on ungulate space-use and home-range behavior.

## Materials and methods

### A note on home-range estimation

Various analytical methods have been developed for quantifying animal home ranges or ‘occurrence distribution’ (OD) based on positional data [44]. Recent developments (reviewed in [45]) highlight the distinction between ‘range distribution’ (the expected lifetime space use; an extrapolation of the positional data) and the ‘occurrence distribution’ (the transient space used during the observation period; an interpolation of the positional data). The range distribution is more compatible with the classical definition of the home range [18], but its quantification requires sufficient positional data to remove the effects of positional and velocity autocorrelations [46], and it does not correspond to where the animal has been but rather where it will be over its lifetime. Because our focus here is on the seasonal dynamics of home range and habitat utilization, we have decided to focus on the occurrence distribution which we quantify using Local Convex Hulls (LoCoH [47, 48]). A LoCoH-based OD is a poor estimator of the range distribution [44], but it has the advantages of making few assumptions and having the capacity to capture hard boundaries (for example, where animals encounter impermeable barriers).

### Study species and telemetry data

We focused on the space-use patterns of two wide-ranging, highly mobile ungulate species of great cultural and economic value in the western United States: mule deer (*Odocoileus hemionus*) and pronghorn (*Antilocapra americana*). Anthropogenic development in the western U.S. threatens both species’ habitats, migration routes, and stopover sites [13, 41, 49–53], causing population declines across species [54, 55]. The state of Utah is home to a wide environmental gradient (Appendix C), giving us the opportunity to study how the space-use decisions of these two ungulate species in response to ALFs differ across different environmental contexts, and to tease apart the effects of ALFs from the effects of the environments they may be associated with.

GPS collar data for both pronghorn and mule deer were provided by the Utah Division of Wildlife Resources (UDWR). Experienced crews from two helicopter companies (Heliwild and Quicksilver) captured the animals using net-guns with no chemical immobilization. UDWR biologists performed processing (e.g. measurements, disease samples, collar deployment, etc.). Biologists either processed the animal at the capture location (for mule deer fawns and all pronghorn) or at a nearby staging site (for mule deer adults). Helicopter chase times did not exceed 5-min and processing times did not exceed 15-min to reduce stress to the animal. Mule deer captures began in December 2012 and pronghorn captures began in December 2017. All captures and processing followed standard UDWR big game capture protocols. Captured animals were fitted with GPS collars programmed to collect fixes at 2-hour intervals.

Both species are partial migrants that may or may not shift ranges in spring and autumn, like other migratory species in temperate regions. As the OD represents the space the animal uses in residency periods, we restricted our temporal window of observations to periods when we were confident the animals would remain resident and not exploring, dispersing, or migrating [37]. We thus chose to subset the data to include only GPS points in the height of winter (the month of February) and summer (the month of July), as neither mule deer nor pronghorn included in our study migrated during these months. Our average data fold (a unique individual-year-season combination) consisted of 280 GPS points (but could be as low as 20 or as high as 2016), corresponding to an average fix rate of 2.5 hours.

### Home range estimation

To delineate occurrence distributions for each individual-season-year, we fitted LoCoH ODs using the ‘amt’ package [56] in R Statistical Software (v4.1.1 [57]). Specifically, we used the fixed number or *k*-LoCoH method, which constructs kernels from each point and *k*-1 of its nearest neighbors [48]. We delineated ODs with nine isopleth levels from 15%-95%, where a 15% isopleth would indicate more intensely used space (the area within which the top 15% of use intensity is concentrated) and a 95% isopleth would indicate less intensely used space [48]. We removed ODs from the analysis that had fewer than 20 GPS points, were tracked for only one day, were smaller than 900-m^2^ (the size of a raster cell, described below), or were outside the extent of the study area. Because LoCoH-based ODs are expected to be sensitive to both the temporal grain and extent of sampling, we weighted each OD by the number of GPS points multiplied by the number of days the animal was tracked during the OD period, normalized. Consequently, in our statistical analysis (described below) we allow better informed ODs to be more influential by giving a higher weight to ODs who had longer tracking periods while also giving less weight to individual-ODs who had the same tracking period but fewer locations [58].

### Environmental data

To evaluate habitat selection, we assembled remote-sensed habitat data—topographic, vegetation, and climate—that would be important predictors to mule deer and pronghorn space use (Table 1). Rasters were projected using a template raster with a 30-m cell resolution and WGS84 UTM12N coordinate reference system to maintain a consistent spatial scale.

**Table 1:** Remotely-sensed habitat data

Environmental Characteristic	Covariate	Product	Spatial-Temporal Grain	Relationship to pronghorn or mule deer ecology	Predictions (all else being equal)
Vegetation	Vegetation phenology (forage)	MOD09Q1 Terra Normalized	Spatial: 250-m Temporal: 8-day	Increased forage quality	Pronghorn: select Mule deer: select
	Herbaceous cover (forage)	Rangeland Analysis Platform (RAP), v3	Spatial: 30-m Temporal: 1-year		
	Tree cover			Increased predation risk for pronghorn. Increased availability of protection from temperature, snow depth, and predation for mule deer. Decreased forage availability.	Pronghorn: avoid Mule deer: seasonal selections
	Shrub cover			Increased forage availability (especially in winter	Pronghorn: select Mule deer: select
Climate	Snow depth	Snow Data Assimilation System (SNODAS)	Spatial: 1-km Temporal: 1-day	Increased costs in movement and thermoregulation energy, decreased forage availability	Pronghorn: avoid Mule deer: avoid
Topography	Elevation	Digital Elevation Model (DEM)	Spatial: 30-m; Temporal: N/A	Increased summer forage availability, increased winter snow depth, increased tree cover	Pronghorn: avoid Mule deer: select in summer
	Roughness			Increased predation risk and movement costs	Pronghorn: avoid Mule deer: avoid

We derived terrain roughness from a digital elevation model (DEM [59]); using the ‘raster’ package [60] in R. We derived herbaceous cover (both species rely heavily on herbaceous vegetation for forage) by summing annual and perennial vegetation cover from the Rangeland Analysis Platform (RAP [61]). We assigned winter vegetation cover values as those from the previous year, assuming no new vegetation growth over the winter. For environmental covariates with sub-monthly temporal scale—snow depth [62] and Normalized Difference Vegetation Index (NDVI [63]);—we used the median value to represent a monthly aggregate per cell. Finally, to quantity forage availability, we multiplied a month’s scaled and median NDVI values (native temporal grain: 16-day) by that month’s herbaceous cover values (native temporal grain: annual). This would attribute the proportion of vegetation greenness that corresponds to herbaceous vegetation, rather than that of tree or shrub cover. We term this product ‘forage’, as higher values correspond to a larger percent cover of green herbaceous vegetation [64].

### Anthropogenic linear feature data

Roads and fences are the dominant ALFs with potential impacts on wildlife in Utah. We accessed GIS layers of road routes from the Utah Geospatial Resource Center (UGRC [65]), categorizing roads into ‘paved’ (interstates, state and U.S. highways, and paved local roads) and ‘unpaved’ (rural, service access, and other unpaved local roads). Roads are easy to detect from satellite imagery, and most are constructed and maintained by government agencies who often keep records of their various attributes, giving us high confidence in our road dataset.

Unlike roads, fences are more difficult to detect from remote imagery, are often constructed by private landowners, and are unregulated [2]. This detection issue is compounded by the dynamic nature of fences, which can be constructed, modified, removed, or deteriorate much faster than roads, necessitating a more frequent mapping effort than other ALFs [2, 41]. In 2020, the UDWR and the Bureau of Land Management (BLM) initiated fence surveys on Utah public lands (R.J., unpublished data); however, these data were spatially limited and not publicly available at the time of this study. The Utah Department of Transportation (UDOT) maintains a publicly available dataset of fences [66], but these data are biased towards fences near highways. Because there is not a readily available, complete dataset of fences in Utah, we created a composite dataset of known and assumed fences that were built on the incomplete fence databases from UDOT and the collaboration with the UDWR and BLM. For the “assumed” fences, we used publicly available GIS layers of land management boundaries (e.g. boundaries between federal, state, tribal, and private entities) [65] and grazing allotments [67, 68] to identify boundaries between rights-holders and assumed that such boundaries would be fenced (sensu [69]). We circle back to the implications of these assumptions in the Discussion.

We rasterized these ALF layers into a road and fence raster where each 30-m cell was denoted by a 0 (meaning no road or no fence was within the cell) or a 1 (meaning a road or a fence was present in the cell). These two rasters were projected using the same template raster as the environmental attributes to maintain spatial consistency.

### Response variables

We calculated the ‘home-range’ size as the area of the ODs 95% isopleth. To quantify the compactness of ‘home-range’ shape, we used the perimeter ($P$) and area ($A$) of the 95% isopleth to calculate a shape index ($SI$):

1 $$SI = {P \over {2\pi \sqrt {{{\rm{A}} \over \pi }} }}$$

where $2\pi \sqrt {{A \over \pi }}$ is the circumference of a circle, thus this equation compares the perimeter of the OD to the circumference of a circle of the same area. Thus, $SI \approx 1$ indicates a more circular, compact shape, and $SI \gg 1$indicates a more elongated, complex shape (Fig. A2).

To quantify use intensity, we rasterized the OD isopleth hulls, assigning each raster cell a value between 1 (low-use intensity) and 9 (high-use intensity) depending on the corresponding isopleth; this would ensure that high-use cells were weighted higher than low-use cells. We then normalized the raster cells so that they sum to 1. To make sure that our results were comparable between individuals, we delineated a standardized “availability” domain for each individual-season-year OD as a 100-km^2^ (the maximum observed home-range area across all ODs) square extent centered around the closest GPS point to the centroid of the OD [37].

Selection or avoidance of a particular attribute is determined by contrasting what is used to what is available on the landscape. Our analytical approach was inspired by Resource Utilization Functions [70, 71]. To quantify the intensity of use of a given attribute, we multiplied the attribute’s raster by the OD raster and summed the cells of the output raster. Because the values of the OD raster sum to 1, this would result in a weighted-average use-intensity of that attribute. To quantify the attribute’s availability, we calculated the average value of the attribute’s raster within each availability domain. To quantify the selection or avoidance of an attribute, we calculated a log-transformed selection ratio ($logSR$) for each attribute ($j$) for each OD:

2 $$logSR = \log \left( {{{use{d_j}} \over {availabl{e_j}}}} \right)$$

where $logSR = 0$ means that the attribute was used at the same intensity as expected based on its availability, indicating no selection nor avoidance. A positive $logSR$ means that the attribute was used more intensely than expected based on its availability, indicating selection. A negative $logSR$ means that the attribute was used less intensely than expected based on its availability, indicating avoidance. The $logSR$ was calculated for each attribute and OD (individual-, season-, and year-specific).

### Modeling

We tested our four hypotheses with linear mixed models (LMM, Table 2) using the ‘lme4’ [72], ‘lmerTest’ [73], and ‘MuMIn’ [74] packages in R. To account for intra-species and -season behavioral variations, we fit every model for each species-season combination. We included an indicator for sex (two-level factor) to account for potential sexual differences in home range properties [21, 75]. We interacted the sex-indicator variable with ALF availabilities to measure how ALF effects vary between sexes. We included individual animal ID as a random effect and weighted the models by the number of GPS points multiplied by the number of days the animal was tracked during the OD period, normalized, allowing better informed ODs to better inform our inference. Besides roads and fences, other habitat characteristics (such as resource availability or landscape configuration) can also affect home range shape or size [19, 29, 75]. To account for this, and to avoid attributing to ALFs variation that is caused by other factors, we included all other environmental attributes as additional predictors in the models evaluating H1. For H2, we only included the environmental predictors that would make movement difficult, thus affecting the OD shape [75], thus we only included terrain roughness and snow depth. For the models evaluating H3 and H4, where the $logSR$ is the response variable, we included the $logSR$ of all other attributes as predictor variables to control for the fact that selecting for one attribute may result in weakening the selection for another, as the animal cannot select for everything at once. The model for pronghorn-winter failed to converge as an LMM due to low sample size, so we removed the random effect and fit this model as a linear model.

**Table 2:** Full models for each hypothesis (all response variables were weighted by the normalized product of the sample size and the sampling duration)

Hypothesis Tested	Response Variable	Full Model
H1	$$\log \left( {Area} \right)$$	$$avai{l_{paved }} + avai{l_{unpaved }} + avai{l_{fence}} + $$ $$avai{l_{elev}} + avai{l_{rough}} + avai{l_{forage}} + avai{l_{shrub}} + avai{l_{tree}} + avai{l_{snow }}_ + $$ $${I_{male}} + $$ $${I_{male}}:\left( {avai{l_{paved}} + avai{l_{unpaved}} + avai{l_{fence}}} \right) + $$ $$nDay{s^*} + (1|animal ID)$$
H2	$$\log {\left( {\log \left( {SI} \right)} \right)^\dag }$$	$$avai{l_{paved}} + avai{l_{unpaved}} + avai{l_{fence}} + $$ $$\left( {avai{l_{rough}} + avai{l_{snow}}} \right) + $$ $${I_{male}} + $$ $${I_{male}}:\left( {avai{l_{paved}} + avai{l_{unpaved}} + avai{l_{fence}}} \right) + $$ $$nDay{s^*} + (1|animal ID)$$
H3	$$logS{R_j}$$ (where $$j$$ is either $$paved roads$$, $$unpaved roads$$, or $$fences$$)	$$avai{l_j} + $$ $${(logS{R_{paved}} + logS{R_{unpaved}} + logS{R_{fence}})^{\rm{\S}}} + $$ $$logS{R_{elev}} + logS{R_{rough}} + logS{R_{forage}} + logS{R_{shrub}} + logS{R_{tree}} + logS{R_{snow}} + $$ $${I_{male}} + {I_{male}}:\left( {avai{l_j}} \right) + (1|animal ID)$$
H4	$$logS{R_j}$$ (where $$j$$ is either $$elevation$$, $$roughness$$, $$forage$$, $$shrub cover$$, $$tree cover$$, or $$snow depth$$)	$$avai{l_j} + $$ $$logS{R_{paved}} + logS{R_{unpaved}} + logS{R_{fence}} + $$ $${(logS{R_{elev}} + logS{R_{rough}} + logS{R_{forage}} + logS{R_{shrub}} + logS{R_{tree}} + logS{R_{snow}})^{\rm{\S}}} + $$ $${I_{male}} + $$ $${I_{male}}:\left( {avai{l_{paved}} + avai{l_{unpaved}} + avai{l_{fence}}} \right) + (1|animal ID)$$

*nDays is a predictor because the number of days an animal was tracked affects the size and shape of the OD

†SI was log-transformed twice because its native range is (1, ∞], thus would be limited by (0, ∞], and so log-transforming it again would map SI to a range of [-∞, ∞]

§$logSR$ would not be a predictor in the same model where it is a response variable

We evaluated the relationships between home range characteristics and focal predictors by calculating model predictions across the range of observed values for each predictor one at a time, while holding all other predictors fixed at their mean value. We calculated 95% confidence intervals around mean estimates using the standard error method [76]. See Fig. A1 for a flow chart of the full methods.

## Results

In total, our analysis consisted of 11,464 ODs: 10,479 from mule deer (54.5% winter and 45.5% summer) and 985 from pronghorn (51.9% winter and 48.1% summer; Fig. A3). Each OD was composed of, on average, 280 GPS points (ranged from 20 to 2016). These ODs were derived from 3425 individuals who had, on average, three ODs each. Of these 3454 individuals, 3105 were mule deer (73% female) and 320 were pronghorn (72% female). Because there were no significant differences in the results between sexes, here we present results on females for simplicity, as females made up most of our sample size. Similarly, while there were differences in effects from unpaved roads compared to paved roads, these differences were minor, so here we present on paved roads and fences only. Results from males and unpaved roads can be found in Appendix B. We have reported all model coefficients in Appendix D.

### Home range size

Pronghorn tended to have larger ODs (0.528 km^2^; SE: 0.50) than mule deer (0.298 km^2^; SE: 0.061). Winter ODs were larger (0.36 km^2^; SE: 0.0607 for deer and 0.67 km^2^; SE: 0.596 for pronghorn) than summer ODs (0.24 km^2^; SE: 0.0604 for deer and 0.38 km^2^; SE: 0.405 for pronghorn).

**Fig. 2:**
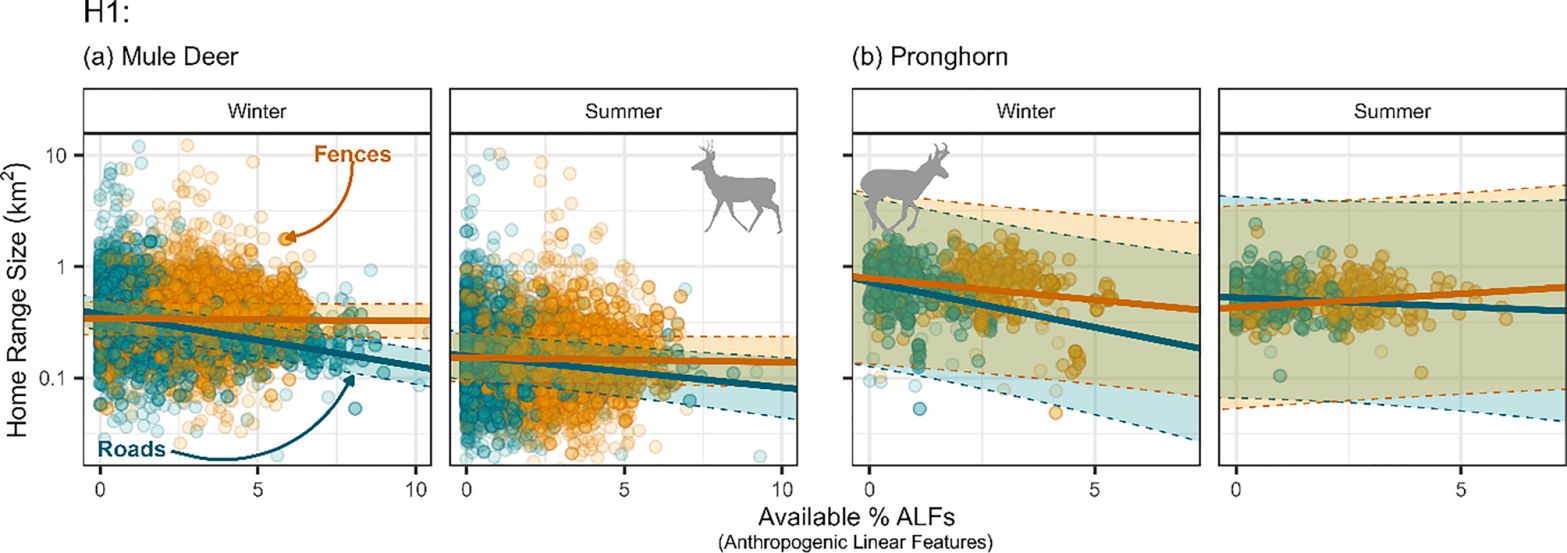
Mule deer (**a**) and pronghorn (**b**) home range size (95% isopleth) in response to paved roads (dark cyan) and fences (orange) density by season (columns). Error bars (dotted lines and transparent fill within the lines) indicate 95% confidence intervals, and point transparency indicates model weights (see Section “Modeling”), where more transparent points indicate less weight in the model and more opaque points indicate more weight in the model

Higher paved road density was significantly correlated with smaller mule deer ODs across seasons (winter: $\beta$ = −0.14, SE: 0.0115; summer: $\beta$ = −0.082, SE: 0.0265; Fig. 2a). Fences, however, had little effect on deer OD size (winter: $\beta$ = −0.0073, SE: 0.0136; summer: $\beta$ = −0.013, SE: 0.0197; Fig. 2a). Pronghorn OD sizes decreased in the winter as paved road density increased ($\beta$ = −0.23, SE: 0.096; Fig. 2b), but otherwise pronghorn OD size did not significantly respond to increasing ALF density (paved roads-summer: $\beta$ = −0.0048, SE: 0.103; fences-winter: $\beta$ = −0.12, SE: 0.0749; -summer: $\beta$ = 0.077, SE: 0.0645).

### Home range shape

OD SIs ranged from 1.07–3.63 and 1.28–3.32 for mule deer and pronghorn, respectively. Mule deer tended to have more compact ODs (SI = 1.9, SE: 5.47) than pronghorn (SI = 2.4, SE: 8.27). Summer ODs tended to be slightly more compact (SI = 1.7, SE: 4.94 and SI = 2.2, SE: 7.78 for deer and pronghorn respectively) than winter ODs for both species (SI = 2.1, SE: 5.99 and SI = 2.6, SE: 8.76 for deer and pronghorn respectively).

**Fig. 3:**
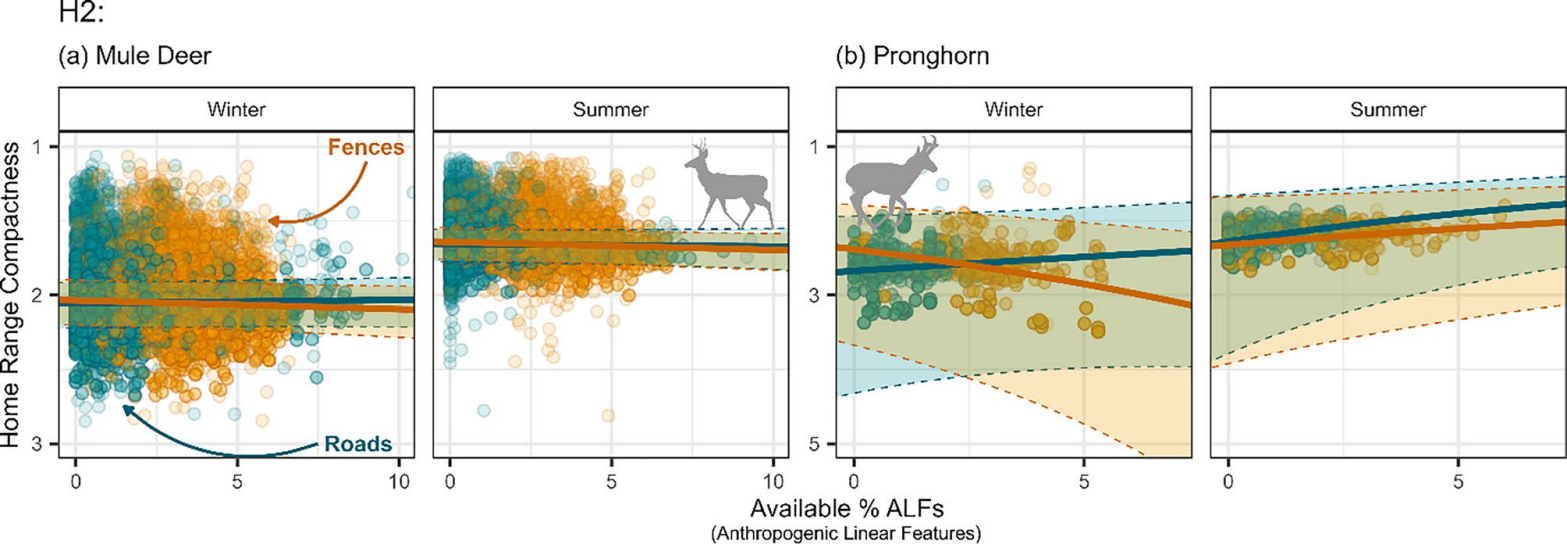
Mule deer (**a**) and pronghorn (**b**) home range shape and compactness (1 indicates a more compact, less complex home range and ≫1 indicates a more complex, less compact home range) in response to paved roads (dark cyan) and fences (orange) density by season (columns). Error bars (dotted lines and transparent fill within the lines) indicate 95% confidence intervals, and point transparency indicates model weights (see Section “Modeling”), where more transparent points indicate less weight in the model and more opaque points indicate more weight in the model

Mule deer OD shapes, overall, did not respond to increasing ALF density. Paved roads and fences did not significantly impact deer OD shapes (paved roads-winter: $\beta$ = −0.0019, SE: 0.00337; -summer: $\beta$ = 0.0023, SE: 0.006237; fences-winter: $\beta$ = 0.0055, SE: 0.00394; -summer: $\beta$ = 0.009, SE: 0.00466; Fig. 3a). Pronghorn OD shapes, however, showed variable responses to ALFs. In the summer, pronghorn ODs tended to become more compact in response to paved roads ($\beta$ = −0.062, SE: 0.0220) while winter OD shapes showed no significant response ($\beta$ = −0.019, SE: 0.0228; Fig. 3b). In contrast, increased fence density was highly correlated with pronghorn OD shapes, where they become more elongated in the winter ($\beta$ = 0.052, SE: 0.0166) and more compact in the summer ($\beta$ = −0.034, SE: 0.0137; Fig. 3b).

### Anthropogenic linear feature selection patterns

**Fig. 4:**
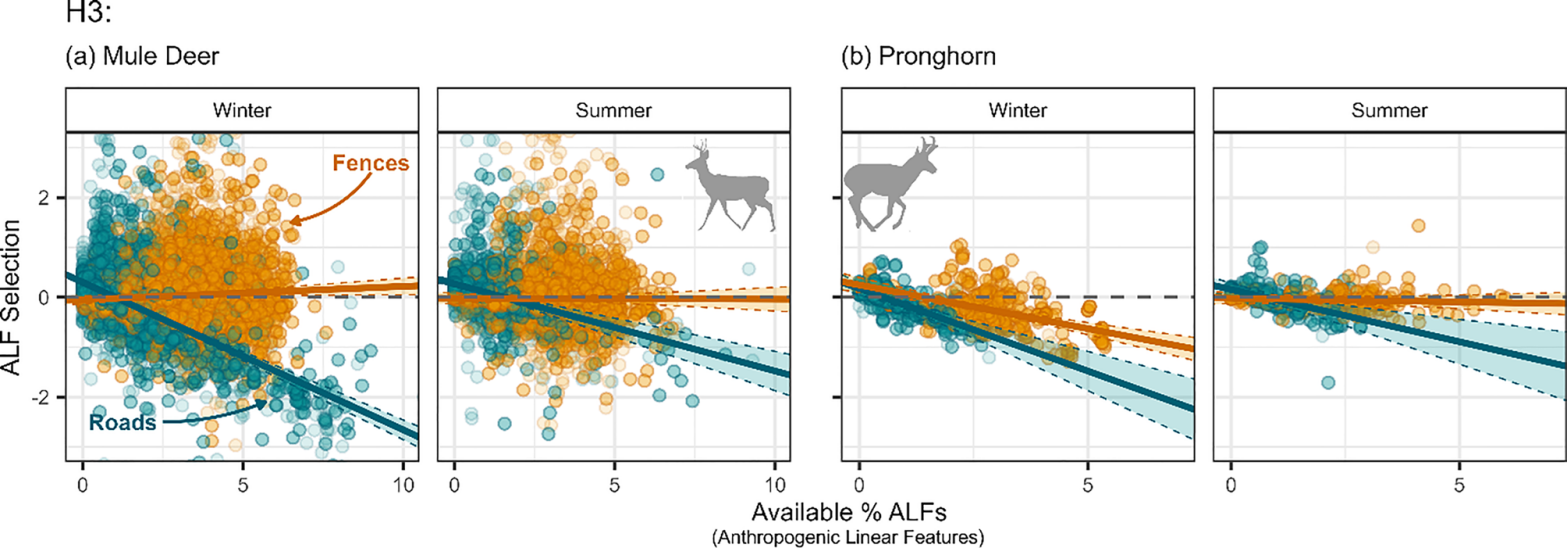
Mule deer (**a**) and pronghorn (**b**) selection patterns for paved roads (dark cyan) and fences (orange) in response to the respective ALF (anthropogenic linear feature) density by season (columns). A negative value indicates avoidance while a positive value indicates selection. Error bars (dotted lines and transparent fill within the lines) indicate 95% confidence intervals, and point transparency indicates model weights (see Section “Modeling”), where more transparent points indicate less weight in the model and more opaque points indicate more weight in the model

Across species and season, individuals tended to increase their avoidance of paved roads as this ALF density increased, particularly in the winter (deer-winter: $\beta$ = −0.371, SE: 0.0142; deer-summer: $\beta$ = −0.219, SE: 0.0270; pronghorn-winter: $\beta$ = −0.42, SE: 0.0609; pronghorn-summer: $\beta$ = −0.26, SE: 0.0667). Mule deer across seasons and pronghorn in summer did not significantly select nor avoid fences and this response did not significantly change in response to fence density (deer-winter: $\beta$ = 0.036, SE: 0.0162; deer-summer: $\beta$ = −0.0027, SE: 0.0225; pronghorn-summer: $\beta$ = −0.02, SE: 0.0283; Fig. 4a,b). However, pronghorn in winter significantly increased their avoidance of fences as fence density increased ($\beta$ = −0.24, SE: 0.0347; Fig. 4b).

### Habitat selection patterns

**Fig. 5:**
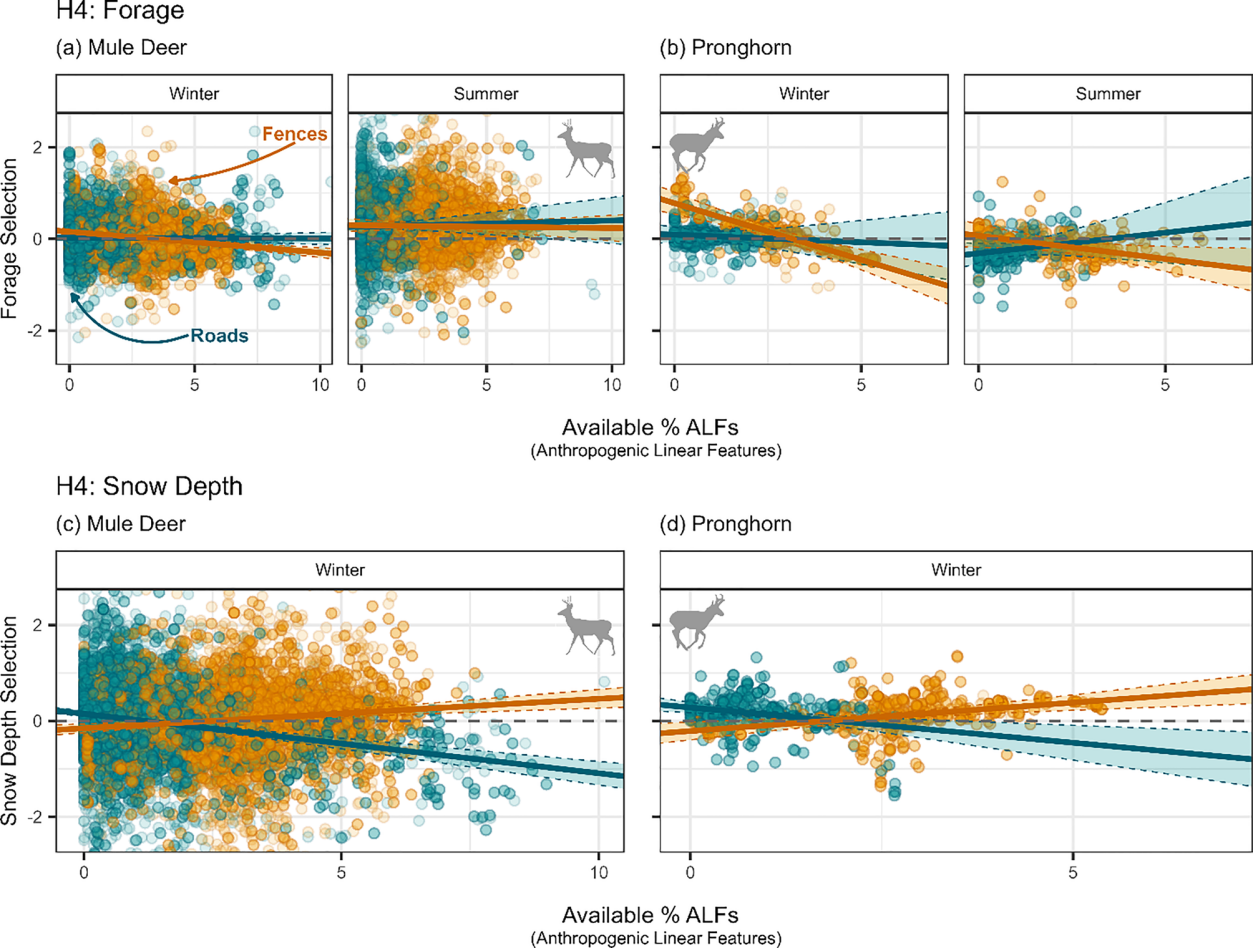
Mule deer (**a,c**) and pronghorn (**b,d**) forage (**a,b**) and snow depth (**c,d**) selection in response to paved roads (dark cyan) and fences (orange) density by season (columns). Error bars (dotted lines and transparent fill within the lines) indicate 95% confidence intervals, and point transparency indicates model weights (see Section “Modeling”), where more transparent points indicate less weight in the model and more opaque points indicate more weight in the model

Increasing ALF density had variable impacts on habitat selection patterns across ALF types, species, and season. Here, we highlight results from forage and snow depth to demonstrate how ALF density affected how animals selected an environmental characteristic we expected animals to select and one we expected animals to avoid, respectively (see Appendix B for other habitat attributes). As expected, mule deer tended to select for forage, and this selection was more significant in the summer (winter: $\beta$ = 0.013, SE: 0.0197; summer: $\beta$ = 0.27, SE: 0.0483). Increased paved road availability had minor effects on mule deer selection for forage in all seasons (winter: $\beta$ = −0.002, SE: 0.00925; summer: $\beta$ = 0.017, SE: 0.0343). Mule deer selection for winter forage decreased as fence density increased ($\beta$ = −0.063, SE: 0.00998), but their summer forage selection patterns did not respond to fences ($\beta$ = −0.0085, SE: 0.02620; Fig. 5a). Against expectations, pronghorn significantly avoided forage in the summer ($\beta$ = −0.25, SE: 0.0835) and did not significantly select nor avoid forage in the winter ($\beta$ = −0.021, SE: 0.07). Pronghorn forage patterns across seasons did not significantly respond to increased paved roads (winter: $\beta$ = −0.041, SE: 0.0739; summer: $\beta$ = 0.11, SE: 0.0102). Fences, however, significantly reduced pronghorn selection for forage in all season, particularly in winter (winter: $\beta$ = −0.34, SE: 0.0563; summer: $\beta$ = −0.14, SE: 0.0628; Fig. 5b).

Mule deer did not significantly select nor avoid snow depth ($\beta$ = 0.025, SE: 0.0213), however, pronghorn, against expectations, slightly tended to select snow depth ($\beta$ = 0.13, SE: 0.0347). Changes in snow depth selection patterns were significantly correlated with increased ALF density for both species, but there were opposite responses to paved roads compared to fences. As paved road density increased, snow depth avoidance increased (mule deer: $\beta$ = −0.16, SE: 0.0176; pronghorn: $\beta$ = −0.18, SE: 0.0563). In contrast, as fence density increased, snow depth selection increased (mule deer: $\beta$ = 0.084, SE: 0.0189; pronghorn: $\beta$ = 0.16, SE: 0.0444; Fig. 5c,d). We wanted to understand if the snow depths that pronghorn and mule deer experienced were significantly different during the study period or not (see Appendix C.2), and we found that, while 2013 was a heavy snow year, the other years in our study period were within one standard deviation of the mean snow depth of previous years, meaning that these snow depths that the pronghorn and mule deer in our study experienced were normal (Fig. C6).

## Discussion

Our study examined the non-lethal effects of anthropogenic linear features on wildlife by comparing seasonal home ranges for two sympatric ungulate species in western North America. Using empirical data, we quantified pronghorn and mule deer home-range properties in response to increased road and fence density, which allowed us to capture deviations from their theoretical optimum. By examining results from multiple hypotheses, we were able to understand how these two ungulate species were impacted by roads and fences in and around their home ranges. We hypothesized that H1) home-range size would increase in response to road density but decrease in response to fence density; H2) home-range shape would become more complex, elongated, and less compact in response to increased ALF density; H3) mule deer and pronghorn would avoid ALFs at a low ALF density, but this avoidance would erode as ALF density increased; H4) selection strength for surrounding habitat attributes would approach zero in response to ALF density (Fig. 1). By looking at the results of each hypothesis as pieces of a whole, our overall findings are: 1) mule deer space-use patterns were heavily affected by paved roads, which reduced their ability to move, and 2) seasonality and snow altered the permeability of fences for pronghorn. These results indicate that roads and fences prevent optimal space-use behavior, possibly resulting in non-lethal but detrimental effects on individual fitness.

### Effects of roads

Mule deer intensely avoided paved roads, particularly as paved road density increased. We further found that mule deer home-range sizes significantly decreased in response to paved road density (12.7% and 7.9% decrease in winter and summer respectively). These finding combined suggest that paved roads are not forcing deer to move more, as we had predicted, but rather are boxing them in and preventing them from moving, thus restricting and shrinking their home-range size. Home range theory predicts that animals should use the smallest space possible to maximize resource intake and minimize energy expenditure [19]. However, as other researchers have noted, it can be difficult to predict how the home range will respond to anthropogenic disturbances and how these responses impact animal fitness [25]. Our results of paved roads decreasing home-range size demonstrates this complexity, as the home range decreasing in this context does not necessarily mean the animal is more efficiently exploiting its environment, but rather, it likely indicates that roads are fully restricting their movement and preventing them from accessing resources. An alternative explanation is that vegetation growing alongside paved roads provides sufficient food subsidies for mule deer to fulfill energy requirements while maintaining a smaller home range; the exploitation of roadside vegetation has been documented in other ungulate species (e.g. [77]). However, in the arid environment of Utah, it is unlikely that mule deer would rely on roadside vegetation to the point of substantially shrinking their space use. A more likely interpretation of the pattern that emerged from our results is that roads are constraining their movement. Even though it did not match the expected deviation from theoretical optimality [19], the pattern we observed suggests that roads have a negative effect on mule deer space use.

While pronghorn also intensely avoided paved roads, this avoidance did not seem to impact pronghorn home ranges in the same way that it did for mule deer. Pronghorn home-range size and shape, overall, did not respond significantly to increased paved road and neither did their forage selection patterns. Perhaps a reason for this lack of strong responses is that there were so few paved roads within pronghorn seasonal ranges to begin with—while some mule deer seasonal ranges had a paved road density as high as 20%, the highest paved road density in a pronghorn seasonal range was 7%. This could indicate that pronghorn avoid paved roads at a coarser scale (2nd-order habitat selection, or where an animal in its geographic range places its home range) than this analysis, leaving their home ranges largely unaffected by road density. Another possibility is that there is a decreased need for people to develop roads in the habitats where pronghorn live that they do not need to avoid them at a coarse scale. This suggests that pronghorn space use at the scale of this analysis (3rd-order habitat selection, or where and what the animal selects within its home range) is less sensitive to paved road development than mule deer.

### Effects of fences

In contrast to paved roads, fences hardly impacted mule deer home ranges. Deer did not strongly avoid fences nor did this selection pattern change as fence density increased, their home-range size and shape did not respond to increased fence density, and their habitat selection patterns were generally unaffected by increased fence density. This indicates that fences may not be a movement impediment for mule deer.

In contrast to mule deer, pronghorn responded more intensely to fences. In the winter, pronghorn significantly avoided fence density, and this avoidance intensely increased with increasing fence density. While this strong avoidance did not seem to affect pronghorn home-range size, their winter home-range shape became more elongated as fence density increased, corroborating our prediction. As a caveat, it is important to mention that the uncertainty around the estimates for pronghorn in winter may have been reduced by the removal of the individual random effect, which we were able to include in all other models.

The differing responses of deer and pronghorn to increased fence density indicate that the permeability of fences is species-specific. Pronghorn tend to crawl under fences rather than jump over [78, though see 79]. As pronghorn rely on their fast speed to avoid predation [80], crossing fences in this way can impair their ability to escape. There is some evidence in the literature to suggest that fences led to increased pronghorn predation, such as the case with [80], who observed coyotes using fences to trap adult pronghorn. In contrast to pronghorn, mule deer can jump over fences. This more agile fence crossing behavior, combined with their general escape behavior—a “stot,” where all four feet touch and leave the ground at the same time and allows mule deer to traverse rough terrain more easily [81]—suggests that mule deer are more able to escape risk and predation through areas with high fence density and other rough terrain. Our findings support those of [79], who found that fences likely pose a lower mortality risk to deer than pronghorn, even though they observed deer crossing fences more frequently. Overall, our findings match our understanding of these two species’ ecology, providing additional evidence that fences constrain pronghorn movement more so than mule deer.

Not only is fence permeability different between species but also between seasons. Pronghorn did not significantly avoid fences in the summer but intensely did so in the winter, and pronghorn home-range composition was only impacted in the winter. Again, we were unable to include a random effect for individual identity in winter models for pronghorn, which may have artificially shrunk confidence intervals around model estimates. Nonetheless, this seasonal difference combined with the result that pronghorn were increasingly unable to avoid snow depth in areas of high fence density suggests that snow accumulation may be altering fence permeability by reducing the space between the ground and the fence and preventing pronghorn from crawling underneath. Compared to other ungulates, pronghorn are more sensitive to cold temperatures and snow [82] and will move hundreds of kilometers to avoid severe winter weather [78, 80]. The ability to avoid deep snow cover is critical for pronghorn winter survival, as demonstrated by a case in 1983 when hundreds of pronghorn starved or froze to death due to snow piling up near fences and preventing them from crossing [83]. Furthermore, we found that the snow depths that pronghorn experienced were within normal ranges compared to previous years (see Appendix C.2), meaning that pronghorn in our study area did not experience extreme snowy or dry winters and we still see a signal of fence avoidance as snow depth increases. As pronghorn in our system also increasingly avoided green forage as fence density increased, areas with high fence density and high snow accumulation could be detrimental for these animals to find resources in the winter when survival is critical.

### Concluding remarks

There has been growing interest in linking animal space-use with its fitness consequences at the individual level and its demographic consequences at the population level. Establishing these links has been challenging, first, due to the lack of availability of simultaneous information on fitness and space use for the same individuals; second, due to the diffuse and cumulative nature of non-lethal effects, which are difficult to attribute to any single driver. The development of multiple hypotheses and the testing of those hypotheses, while controlling for alternative drivers, has recently provided important breakthroughs—for instance, in measuring non-consumptive effects of predators on prey [84]. Similarly, our study shows that non-lethal effects of anthropocentric structures can be detected and quantified by simultaneously evaluating multiple hypotheses pertaining to different metrics of space use while controlling for other factors.

While we did not measure fitness, we relied on a large body of theoretical literature that ties the properties of animal home ranges to their fitness consequences. Despite the importance of this theoretical connection, home ranges are often used as a purely descriptive tool to quantify the patterns of animal space use. Our study provides an example of how the theoretical underpinnings of the home range concept can be leveraged to make inferences beyond description and begin to answer elusive questions about the effects of chronic stressors on wildlife populations. In this study, we focused on non-lethal effects of roads and fences; however, a similar approach can be used to evaluate non-lethal effects of other factors that are expected to affect animal home-ranging behavior—such as drought or altered fire regimes—so long as concurrent drivers of animal space use are identified and measured.

While we presented responses at the population level, our results showed wide individual variability, even among groups of the same species and sex and in the same season. This variability is not surprising and should be expected, because home range size, shape, and selection are all influenced by a wide variety of factors, both extrinsic and intrinsic—such as resource availability in a certain year or seasons, landscape configuration, individual body size, and differences in behavior. This multicausality inevitably introduces noise in our estimates of the effect of any single factor on home range characteristics. Nonetheless, we were able to extract signals from the noise and detect significant effects of ALFs on home range size, shape, and selection for some species-season combinations.

Furthermore, these wide variations in space-use responses could lead us to conclude that, at the population level, these animals respond to roads and fences in a certain way, while individuals within different sub-populations may respond differently. Further research should focus on disentangling drivers of this variability. One study, for example, found that migrating compared to resident pronghorn exhibited differing selection habits for fences and roads [50], indicating that migration status can affect habitat and ALF selection. Another possibility for this variation could be because of the wide variation in climate and topography in Utah (see Appendix C). These additional behavioral, intrinsic, and extrinsic factors could provide more nuance to the wide individual variation we observed.

A promising direction for future research is to quantify responses of animals to ALFs depending on their specific attributes, beyond coarse characterizations such as paved versus unpaved roads, e.g. lane width, traffic volume, fence height, fence type, etc. These attributes likely modulate behavioral responses to ALFs, and a fine-scale examination of ALF effects on animal movement and space use would benefit from adding these attributes to an analysis [50]. Furthermore, our fence data, as it was a combination of known and assumed fences, might not have fully represented (or possibly misrepresented) actual fences on the landscape. Fences are one of the most expansive anthropogenic features on the landscape, often at higher densities than roads [2, 9, 49], and yet comprehensive and accurate data on their locations is lacking; having a comprehensive census of known fences would provide a more accurate understanding of wildlife responses to fences.

This research not only expands the theoretical literature of animal home ranges and their response to ALFs but also offers some practical strategies for wildlife managers to take when mitigating ALF impacts on their target species. Our findings highlight the species- and season-specific nature of ALF impacts, showing that mitigation strategies on one linear feature for one species may be ineffective for another species. The result that pronghorn are more sensitive to fences in the winter than the summer shows the seasonal effects of ALFs and demonstrates that regions with deep snow cover may need to make different mitigation efforts—such as lay-down fences [85]—than regions that do not receive as much snow. More broadly, simultaneously looking at how anthropogenic structures affect multiple aspects of an animal’s space use can provide detailed information in support of management decisions that we could not get from looking at various aspects in isolation.

## Electronic supplementary material

Below is the link to the electronic supplementary material.

Supplementary Material 1

Supplementary Material 2

## Data Availability

Home range data that support the findings of this study have been deposited onto Dryad. This dataset is currently only available to reviewers at the following link: http://datadryad.org/share/LINK_NOT_FOR_PUBLICATION/sMArt0wymrDZXKZCkfZ1Wb3SuYRsXD_TbP8Lev0tI8o
