# Supplementary material for: The impacts of anthropogenic linear features on the space-use patterns of two sympatric ungulates
Source: Mov Ecol. 2026 Feb 18;14:19. doi: 10.1186/s40462-026-00628-y (PMC13019967; doi:10.1186/s40462-026-00628-y)
Supplement: Supplementary file 1 — Supplementary Material 1 [file 40462_2026_628_MOESM1_ESM.docx]

# Appendix A: Supplementary materials


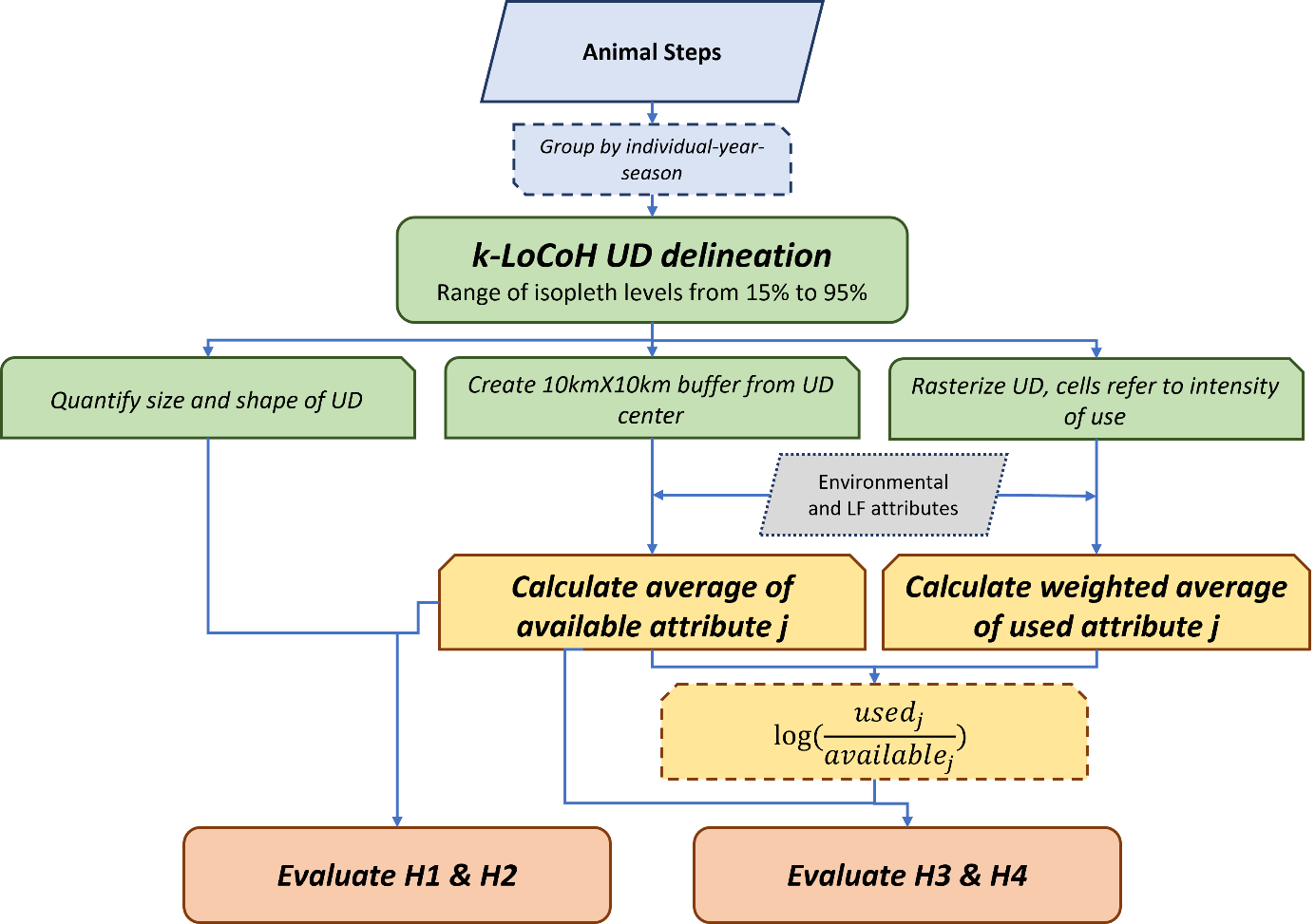


**Figure A1:** Flowchart of methods.


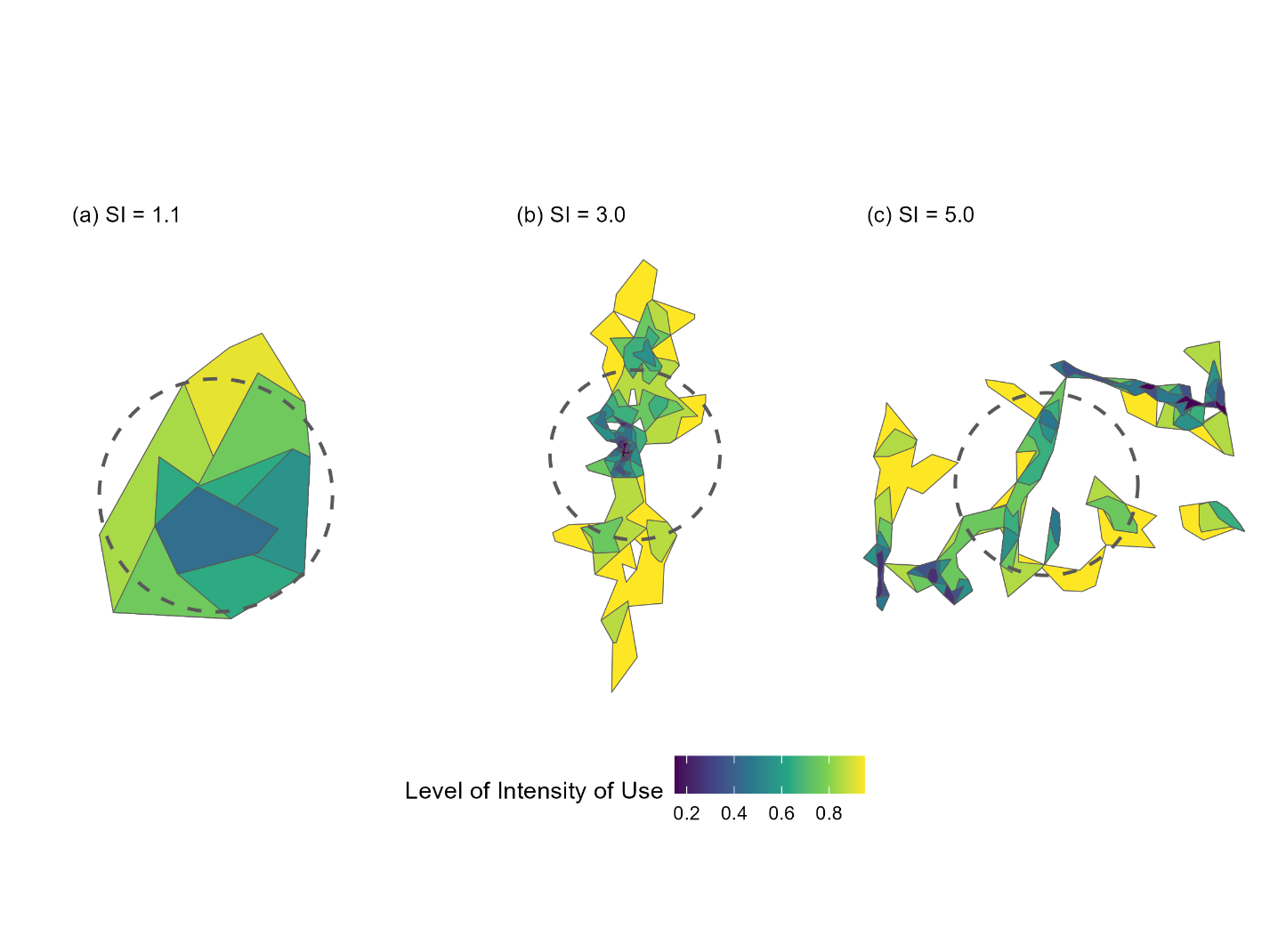


**Figure A2**: A sample of three ODs of differing compactness to show a shape index of around (a) 1, (b) 3, and (c) 5. The darker areas are more intensely used areas while the lighter areas are less intensely used areas. The dashed circle represents the same area as the OD to show the OD shape’s deviation from optimality.


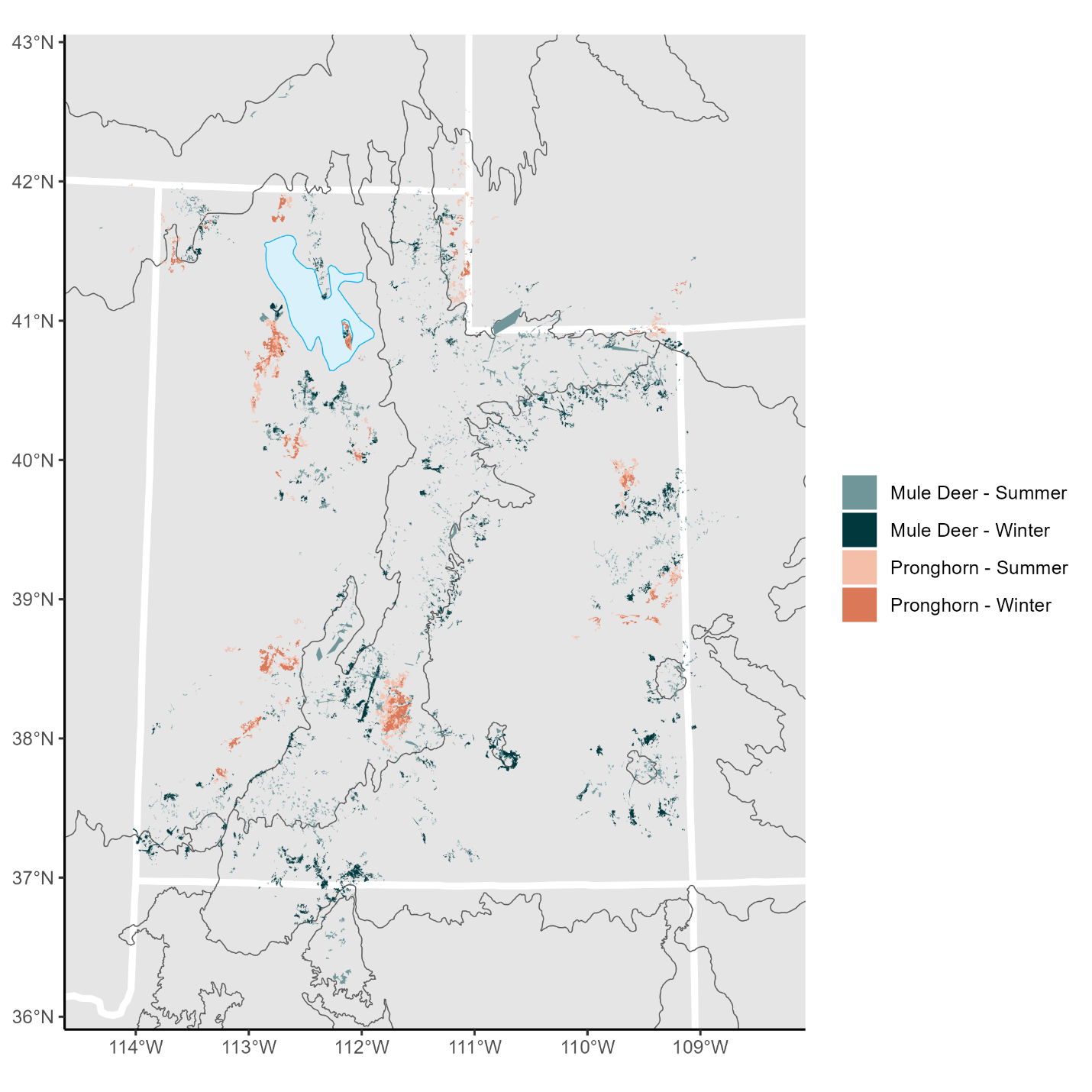


**Figure A3**: Mule deer and pronghorn summer (light blue and light orange, respectively) and winter (dark blue and dark orange, respectively) ODs within the study area. The white boundaries delineate the states, and the grey boundaries delineate the Level III ecoregions.

**Table A1:**

| Response | Species | Season | R-Squared | Pearson’s correlation coefficient |
| --- | --- | --- | --- | --- |
| log(Area) | Mule deer | Winter | 0.254 | 0.523 |
|  |  | Summer | 0.164 | 0.462 |
|  | Pronghorn | Winter | 0.398 | 0.675 |
|  |  | Summer | 0.434 | 0.680 |
| log(log(Shape)) | Mule deer | Winter | 0.119 | 0.263 |
|  |  | Summer | 0.126 | 0.240 |
|  | Pronghorn | Winter | 0.271 | 0.177 |
|  |  | Summer | 0.251 | 0.675 |
| logSR Paved Roads | Mule deer | Winter | 0.286 | 0.502 |
|  |  | Summer | 0.160 | 0.484 |
|  | Pronghorn | Winter | 0.434 | 0.670 |
|  |  | Summer | 0.004 | -0.128 |
| logSR Unpaved Roads | Mule deer | Winter | 0.160 | 0.400 |
|  |  | Summer | 0.047 | 0.236 |
|  | Pronghorn | Winter | 0.090 | 0.298 |
|  |  | Summer | -0.002 | 0.220 |
| logSR Fence | Mule deer | Winter | 0.047 | 0.263 |
|  |  | Summer | -0.002 | -0.007 |
|  | Pronghorn | Winter | 0.153 | 0.432 |
|  |  | Summer | 0.096 | 0.431 |
| logSR Forage | Mule deer | Winter | 0.134 | 0.342 |
|  |  | Summer | 0.261 | 0.510 |
|  | Pronghorn | Winter | 0.410 | 0.542 |
|  |  | Summer | 0.238 | 0.691 |
| logSR Snow Depth | Mule deer | Winter | 0.360 | 0.597 |
|  | Pronghorn | Winter | 0.373 | 0.627 |
| logSR Elevation | Mule deer | Winter | 0.465 | 0.700 |
|  |  | Summer | 0.235 | 0.433 |
|  | Pronghorn | Winter | 0.505 | 0.713 |
|  |  | Summer | 0.282 | 0.534 |
| logSR Roughness | Mule deer | Winter | 0.357 | 0.624 |
|  |  | Summer | 0.252 | 0.473 |
|  | Pronghorn | Winter | 0.571 | 0.760 |
|  |  | Summer | 0.399 | 0.614 |
| logSR Shrub Cover | Mule deer | Winter | 0.310 | 0.544 |
|  |  | Summer | 0.287 | 0.546 |
|  | Pronghorn | Winter | 0.250 | 0.544 |
|  |  | Summer | 0.522 | 0.746 |
| logSR Tree Cover | Mule deer | Winter | 0.299 | 0.533 |
|  |  | Summer | 0.421 | 0.664 |
|  | Pronghorn | Winter | 0.958 | 0.980 |
|  |  | Summer | 0.839 | 0.915 |

# Appendix B: Results for males, unpaved road effects, and selection patterns for other habitat attributes.

## B.1 Home Range Size

Like their female counterparts, increased paved road density was significantly correlated with smaller male mule deer ODs (winter: -0.157±0.05; summer: -0.139±0.07), and their OD sizes did not significantly respond to increased fence density (winter: -0.011±0.05; summer: -0.036±0.07; Fig. B1a). Male pronghorn winter ODs also significantly decreased in size as paved road density increased (-0.323±0.26), but otherwise male pronghorn OD sizes did not show a significant response to increased paved road (summer: -0.068±0.31) nor fence density (winter: 0.107±0.27; summer: -0.087±0.22; Fig. B1b).


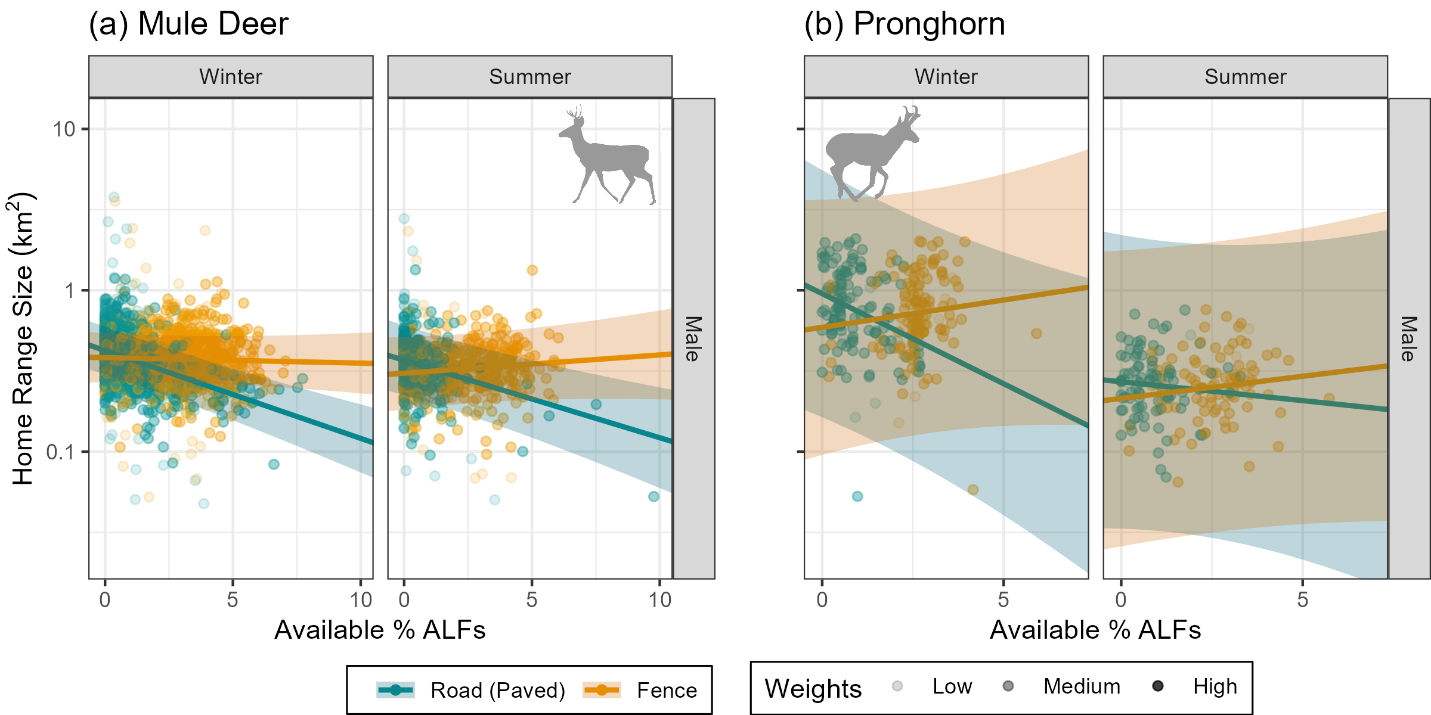


**Figure B1**: Male mule deer (a) and pronghorn (b) OD size (95% isopleth) in response to paved road (dark cyan) and fence (orange) density by season (columns).

Mule deer OD sizes, across sex and season, did not significantly respond to increased unpaved roads (female-winter: -0.004±0.03, female-summer: -0.052±0.04; male-winter: 0.001±0.05, male-summer: -0.015±0.08; Fig. B2a). Female pronghorn OD sizes did not respond to increased unpaved road density in either winter (-0.031±0.12) nor summer (0.119±0.12), and neither did male-winter pronghorn ODs (0.143±0.21). However, male-summer pronghorn ODs slightly increased in size as unpaved road density increased (0.331±0.21; Fig. B2b).


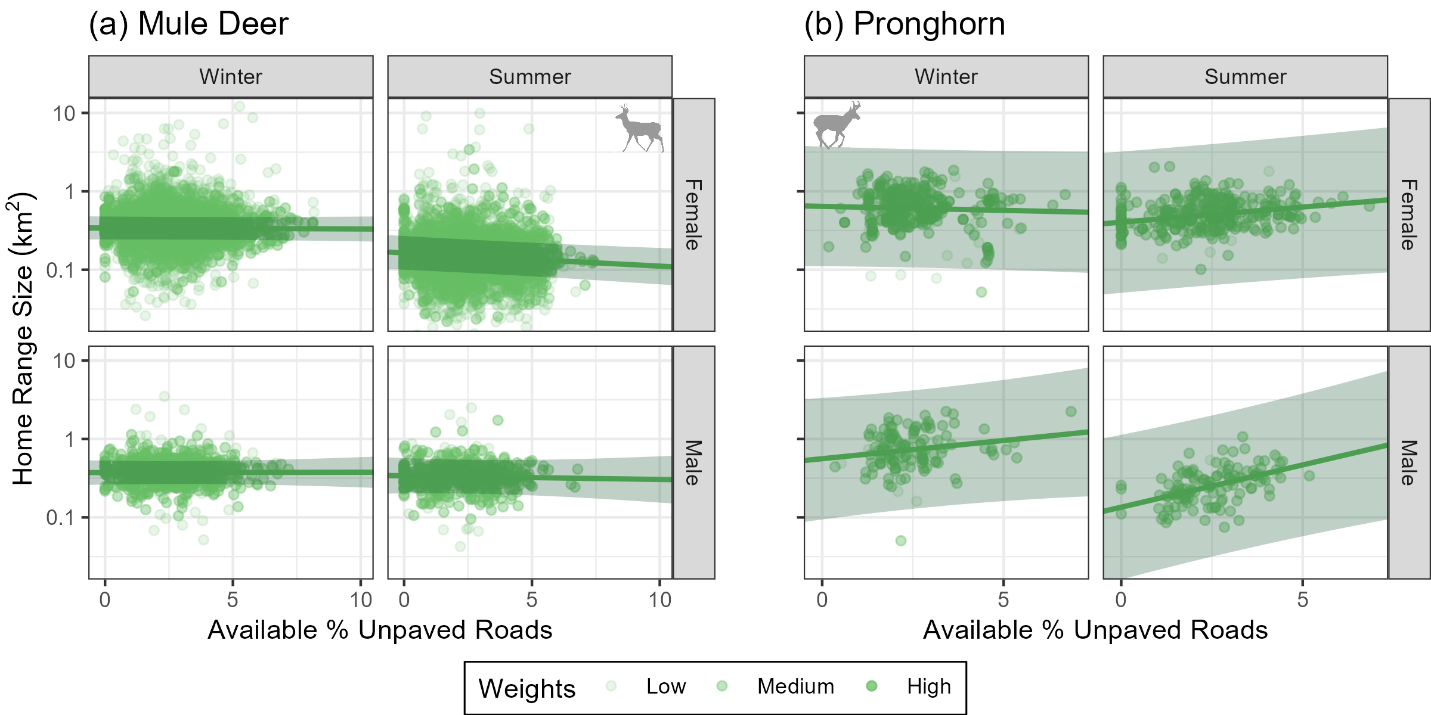


**Figure B2:** Mule deer (a) and pronghorn (b) OD size (95% isopleth) in response to unpaved road density by sex (rows) and season (columns).

## B.2 Home Range Shape

Across season and species, male OD shapes neither significantly responded to paved roads (deer-winter: -0.014±0.01, deer-summer: 0.008±0.02; pronghorn-winter: -0.030±0.06, pronghorn-summer: 0.003±0.07) nor fences (deer-winter: -0.009±0.01, deer-summer: 0.010±0.02; pronghorn-winter: 0.016±0.07, pronghorn-summer: -0.022±0.05; Fig. B3). While female deer ODs in both seasons significantly became more compact as unpaved road density increased (winter: -0.015±0.01; summer: -0.010±0.01; Fig. B4a), these responses were minor. Otherwise, OD shapes did not significantly respond to increasing unpaved roads (male deer-winter: -0.013±0.02, male deer-summer: -0.012±0.02; female pronghorn-winter: -0.019±0.03, female pronghorn-summer: -0.003±0.03; male pronghorn-winter: -0.004±0.05, male pronghorn-summer: 0.024±0.04; Fig. B4).


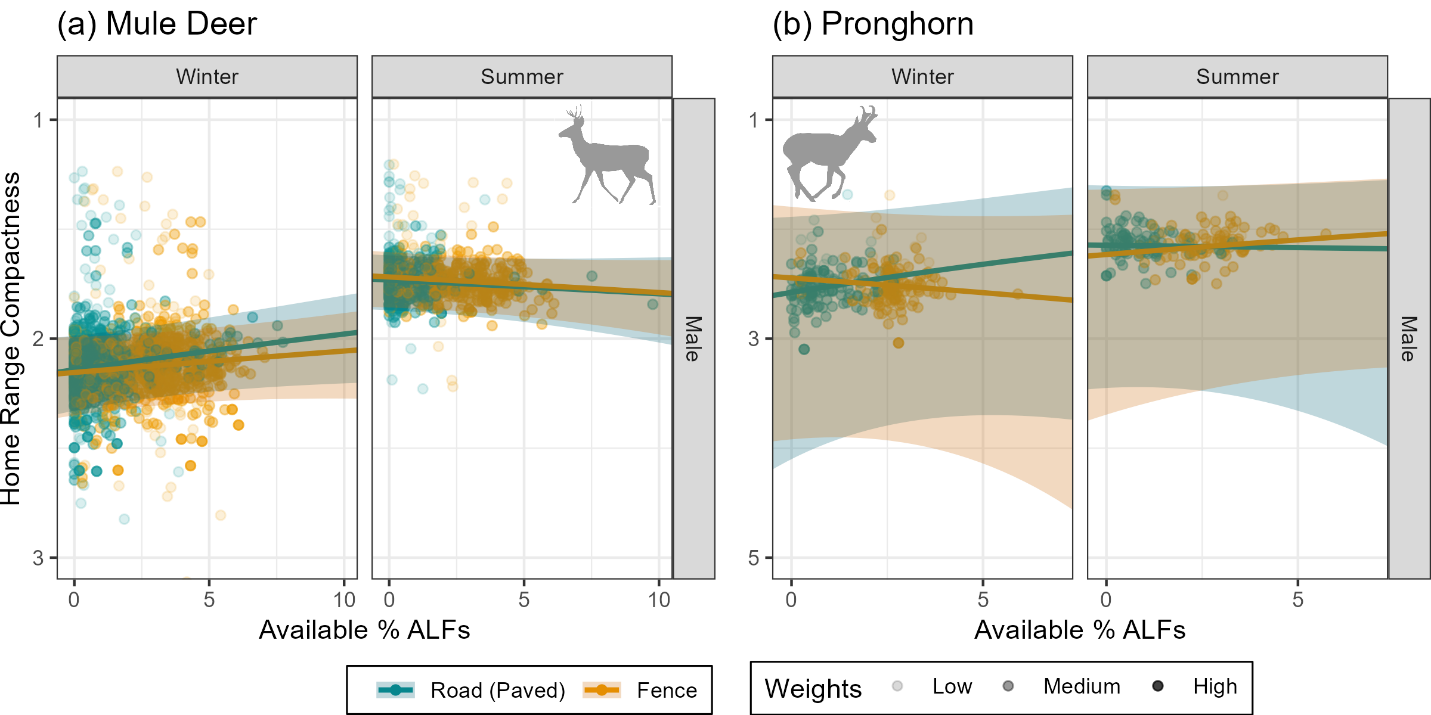


**Figure B3:** Male mule deer (a) and pronghorn (b) OD compaction (1 indicates a more compact OD and ≫1 indicates a more complex OD) in response to paved road (dark cyan) and fence (orange) density by season (columns).


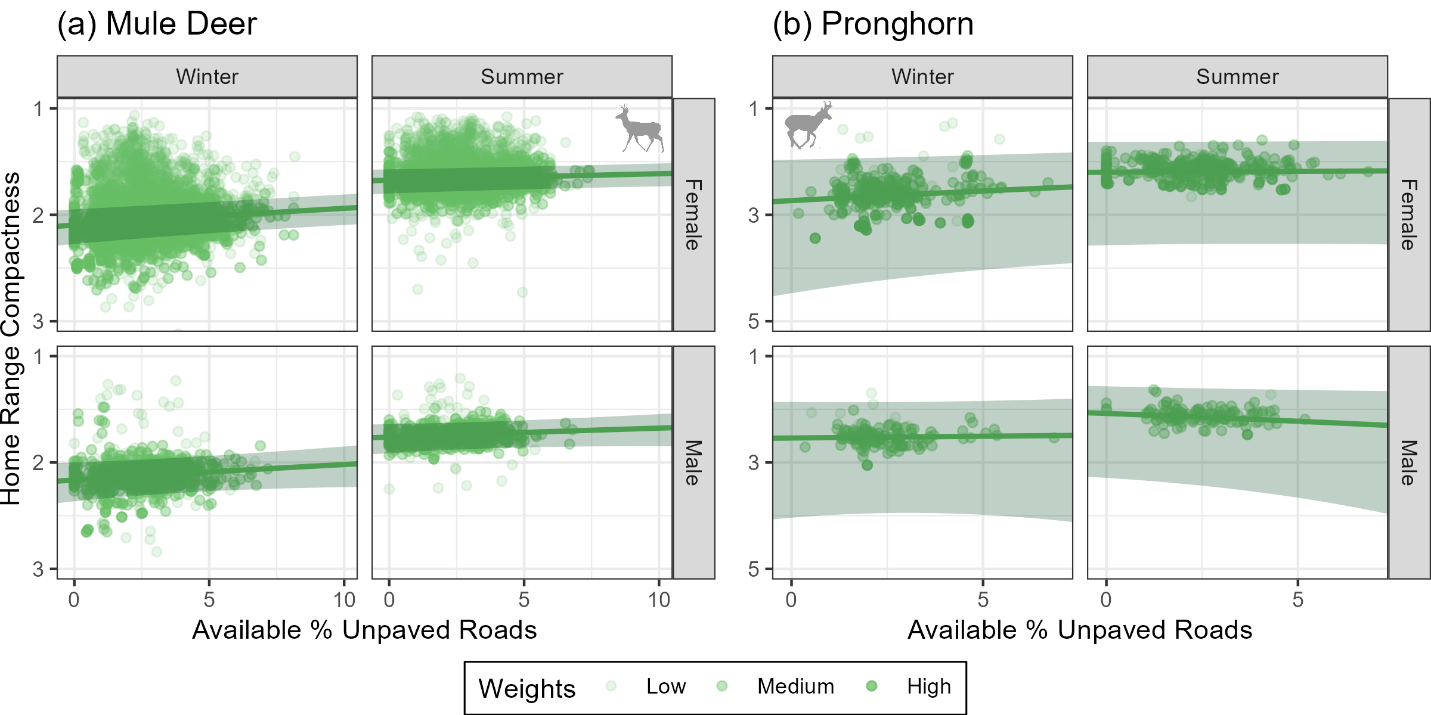


**Figure B4:** Male mule deer (a) and pronghorn (b) OD compaction in response to unpaved road density by sex (rows) and season (columns).

## B.3 Anthropogenic Linear Feature Selection Patterns

Like their female counterparts, male mule deer and pronghorn significantly avoided paved roads (except for male pronghorn in summer: -0.171±0.22), and this avoidance intensely increased as paved road density increased (deer-winter: -0.421±0.06; deer-summer: -0.113±0.08; pronghorn-winter: -0.479±0.22; Fig. B5). For the most part, neither mule deer nor pronghorn significantly selected nor avoided unpaved roads and this did not change as unpaved road density increased (female deer-winter: 0.007±0.03; male deer-winter: -0.047±0.06; male deer-summer: -0.077±0.09; female pronghorn-winter: 0.015±0.06; female pronghorn-summer: -0.052±0.09; male pronghorn-winter: -0.089±0.011; male pronghorn-summer: 0.094±0.17; Fig. B6). The exception is female deer in summer, who slightly avoided unpaved roads as unpaved road density increased, but this avoidance was not intense (-0.070±0.04; Fig. B6a).


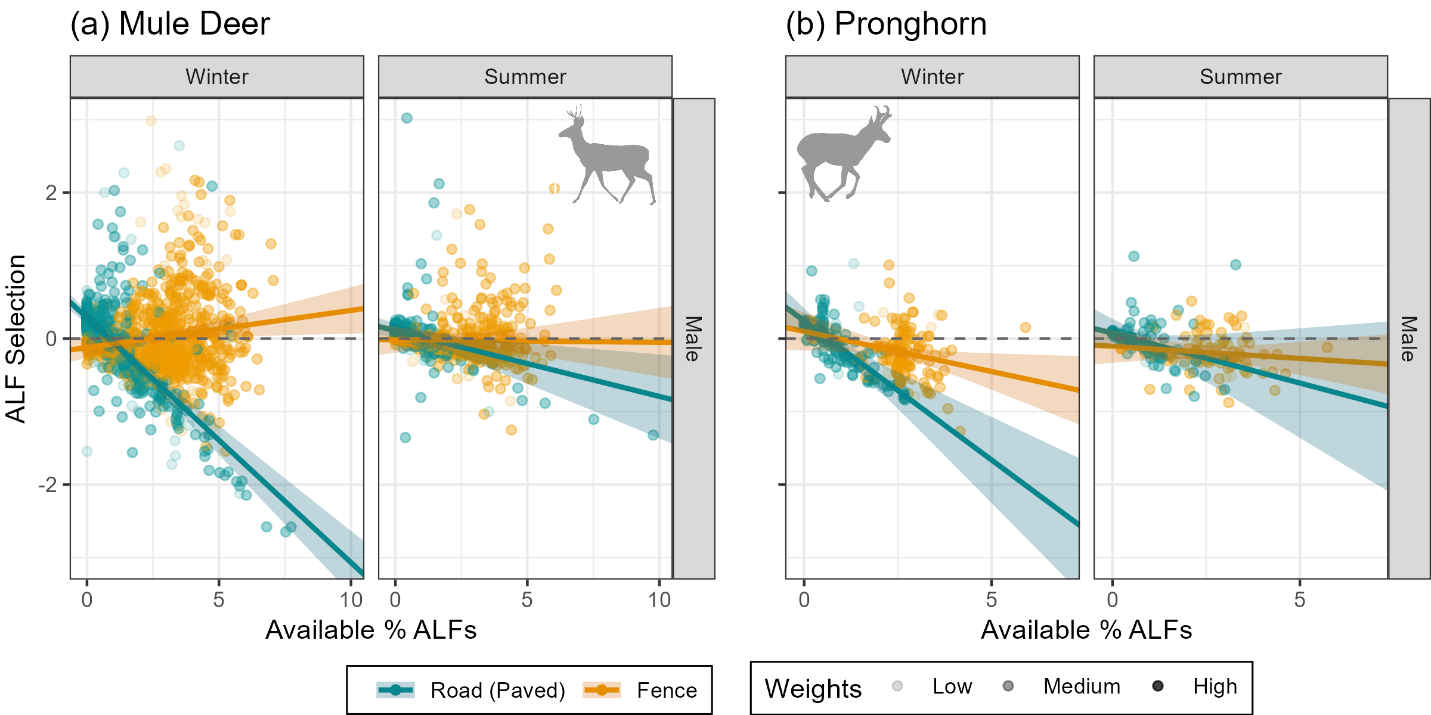


**Figure B5:** Male mule deer (a) and pronghorn (b) selection patterns for paved roads (dark cyan) and fences (orange) in response to the respective ALF density by season (columns).


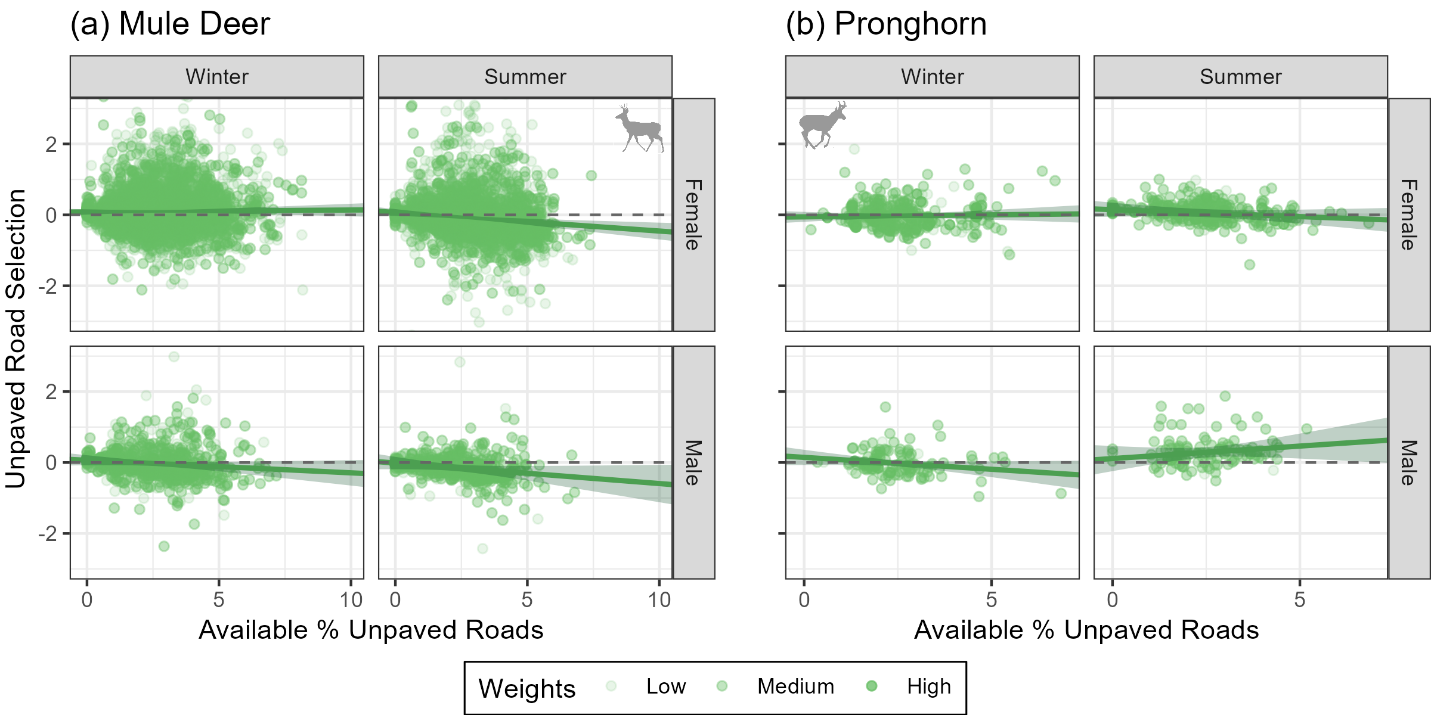


**Figure B6:** Mule deer (a) and pronghorn (b) selection patterns for unpaved roads in response to unpaved road density by sex (rows) and season (columns).

## B.4 Habitat Selection Patterns

As expected, mule deer tended to select for elevation in the summer (female: 0.124±0.05; male: 0.465±0.1) and avoided elevation in the winter (female: -0.139±0.03; male: -0.122±0.06). Increased paved road density was significantly correlated with mule deer increasing their avoidance of elevation (female-winter: -0.100±0.02; male-winter: -0.111±0.04; female-summer: -0.401±0.05; male-summer: -0.199±0.08), causing mule deer in the summer to switch their elevation selection patterns from selection to avoidance (Fig. B7a). Increased fence density was significantly correlated with mule deer increasing their elevation avoidance in the winter (except for males: -0.004±0.05; females: -0.041±0.02), but in the summer mule deer significantly increased their elevation selection as fence density increased (female: 0.111±0.04; male: 0.229±0.08). Unpaved roads were not significantly correlated with changes in mule deer elevation selection patterns (female-winter: 0.041±0.02; male-winter: 0.001±0.05; female-summer: -0.025±0.04; male-summer: -0.179±0.09). While female-winter and male-summer patterns significantly responded to unpaved road density, these responses were muted (Fig. B8a).

Pronghorn significantly avoided elevation in the winter (female: -0.095±0.04; male: -0.057±0.05) but showed no significant avoidance nor selection in the summer (female: 0.075±0.08; male: -0.047±0.10). Pronghorn selection patterns for elevation did not respond significantly or intensely to increased ALF density. Female pronghorn in the summer increased their elevation avoidance as paved road density increased (-0.191±0.11), but otherwise paved roads did not significantly affect pronghorn selection for elevation (female-winter: -0.033±0.06; male-winter: -0.079±0.09; male-summer: -0.108±0.16; Fig. B7b). While pronghorn significantly increased their avoidance of elevation as fence density increased (except for female-summer: 0.025±0.07), these responses were not strong (female-winter: -0.094±0.05; male-winter: -0.095±0.09; male-summer: -0.145±0.12; Fig. B7b). Apart from female-winter (-0.054±0.04), pronghorn selection for elevation did not significantly respond to increased unpaved road density (male-winter: -0.054±0.06; female-summer: -0.053±0.06; male-summer: -0.017±0.11; Fig. B8b).


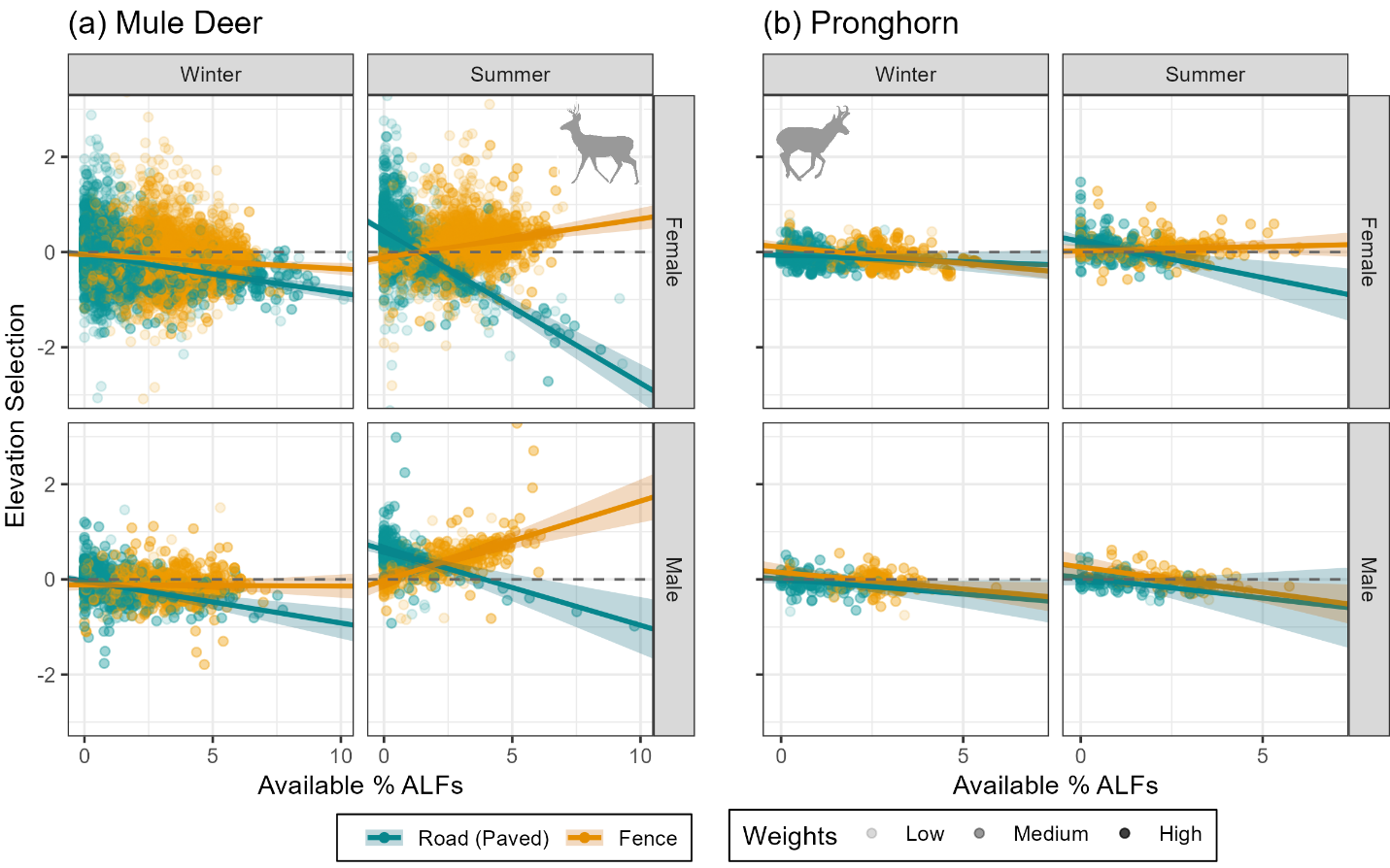


**Figure B7:** Mule deer (a) and pronghorn (b) selection for elevation in response to paved road (dark cyan) and fences (orange) density by sex (rows) and season (columns).


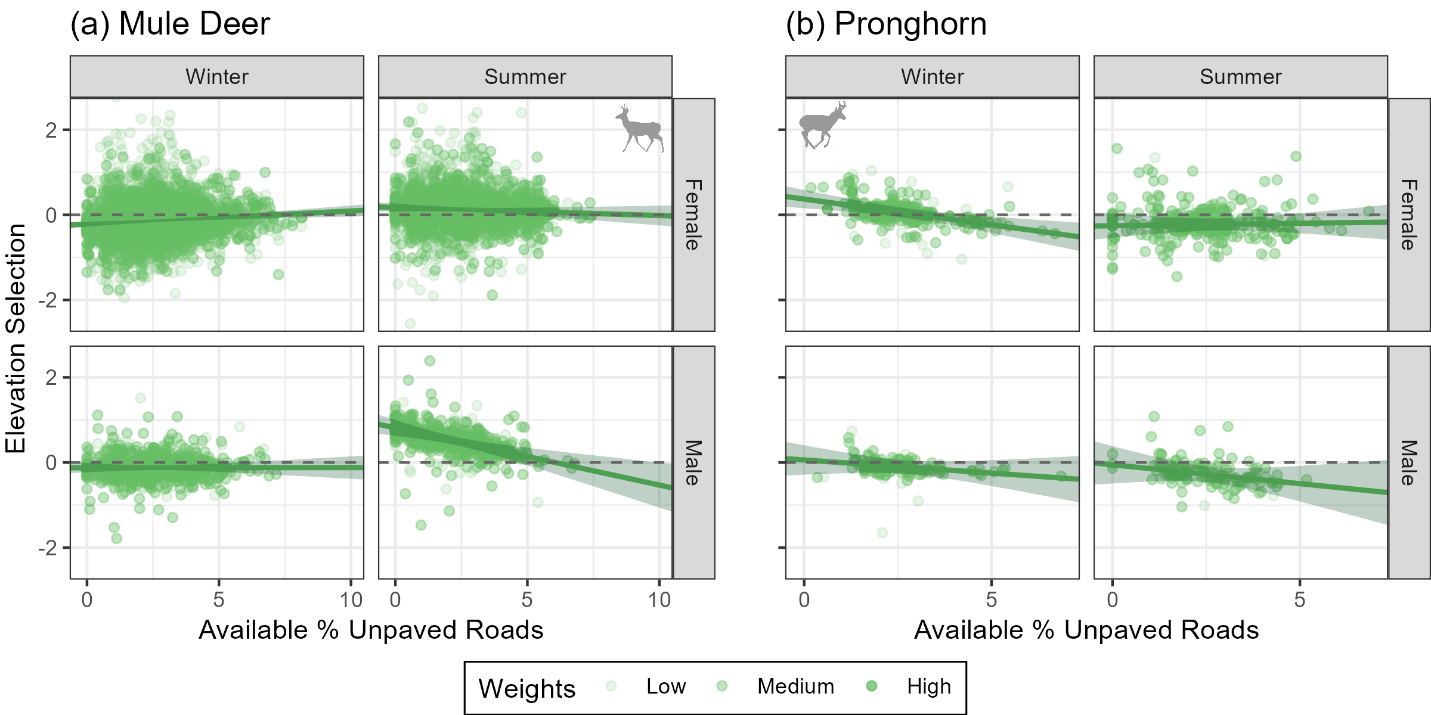


**Figure B8**: Mule deer (a) and pronghorn (b) selection for elevation in response to unpaved road density by sex (rows) and season (columns).

Interestingly, female mule deer significantly selected for roughness in the winter (0.082±0.04) but not in the summer (-0.004±0.07), while male mule deer significantly avoided roughness in the summer (-0.114±0.09) but not in the winter (0.002±0.04). Increased paved road density was significantly correlated with increased roughness selection for female mule deer in the winter (0.042±0.03) and increased avoidance for female mule deer in the summer (-0.069±0.05), while male mule deer selection patterns for roughness did not significantly respond to paved road density (winter: -0.036±0.06; summer: -0.032±0.07; Fig. B9a). In the winter, mule deer of both sexes increased their avoidance of roughness as fence density increased (female: -0.085±0.03; male: -0.104±0.06), while their summer selection patterns for roughness did not significantly respond to fences (female: 0.028±0.04; male: 0.012±0.07). Overall, mule deer selection or avoidance of roughness did not respond to increased unpaved road density, except for females in the summer (-0.073±0.04), however this response was muted (female-winter: 0.001±0.03; male-winter: -0.013±0.06; male-summer: -0.035±0.08; Fig. B10a).

Pronghorn, in contrast, across sex and season, significantly avoided roughness (female-winter: -0.360±0.14; male-winter: -0.441±0.15; female-summer: -0.530±0.12; male-summer: -0.410±0.13). However, these selection patterns did not significantly respond to paved roads (female-winter: 0.030±0.0.9; male-winter: 0.038±0.13; female-summer: 0.024±0.09; male-summer: -0.128±0.14) nor fences (female-winter: 0.043±0.07; male-winter: -0.083±0.13; female-summer: -0.014±0.06; male-summer: 0.008±0.10; Fig. B9b). While changes in pronghorn selection patterns for elevation were significantly correlated with increased unpaved road density, these responses were muted and did not cause their selection patterns to switch from avoidance to selection (female-winter: 0.073±0.05; male-winter: -0.016±0.09; female-summer: -0.059±0.05; male-summer: -0.115±0.10; Fig. B10b)


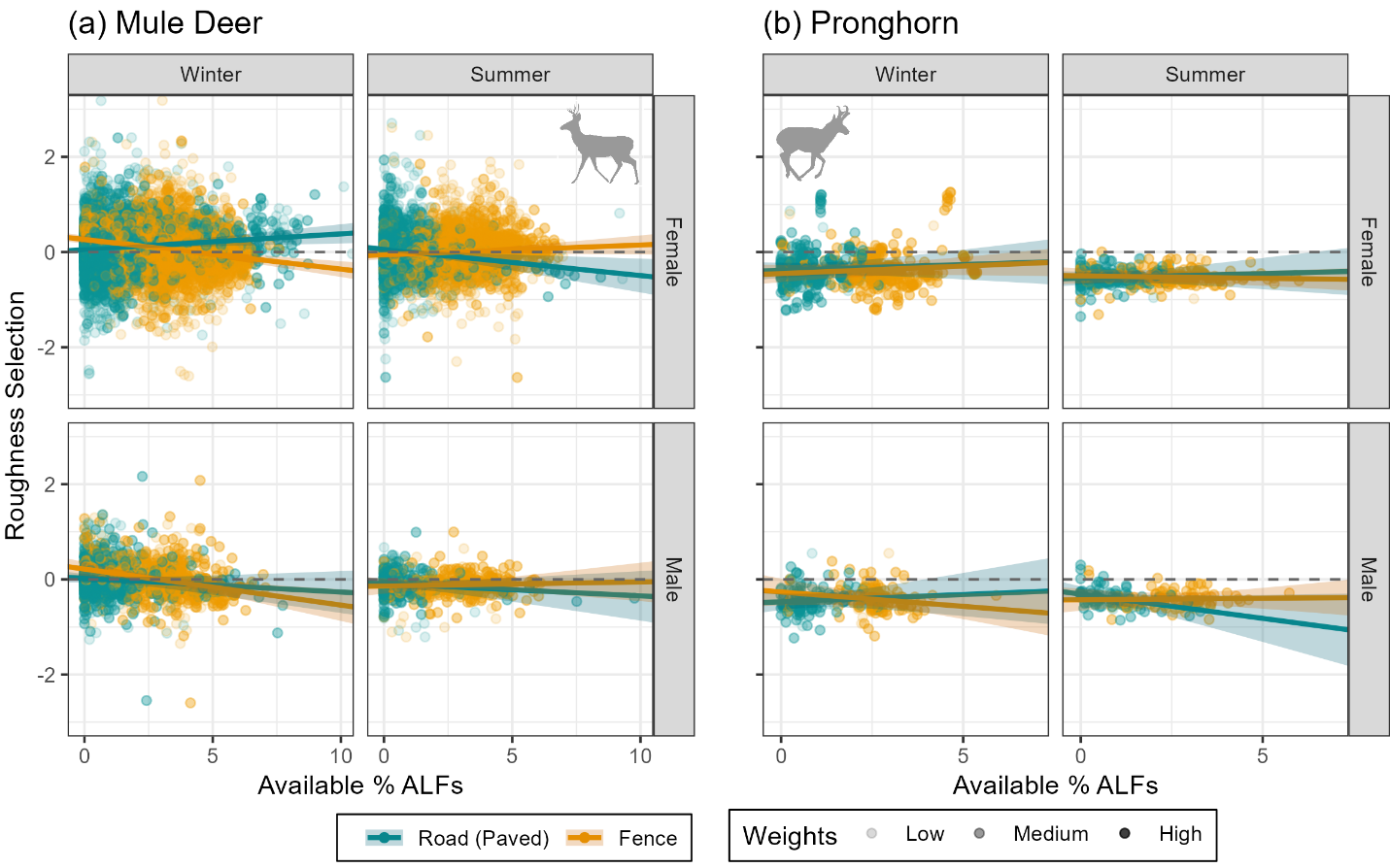


**Figure B9:** Mule deer (a) and pronghorn (b) selection for roughness in response to paved road (dark cyan) and fences (orange) density by sex (rows) and season (columns).


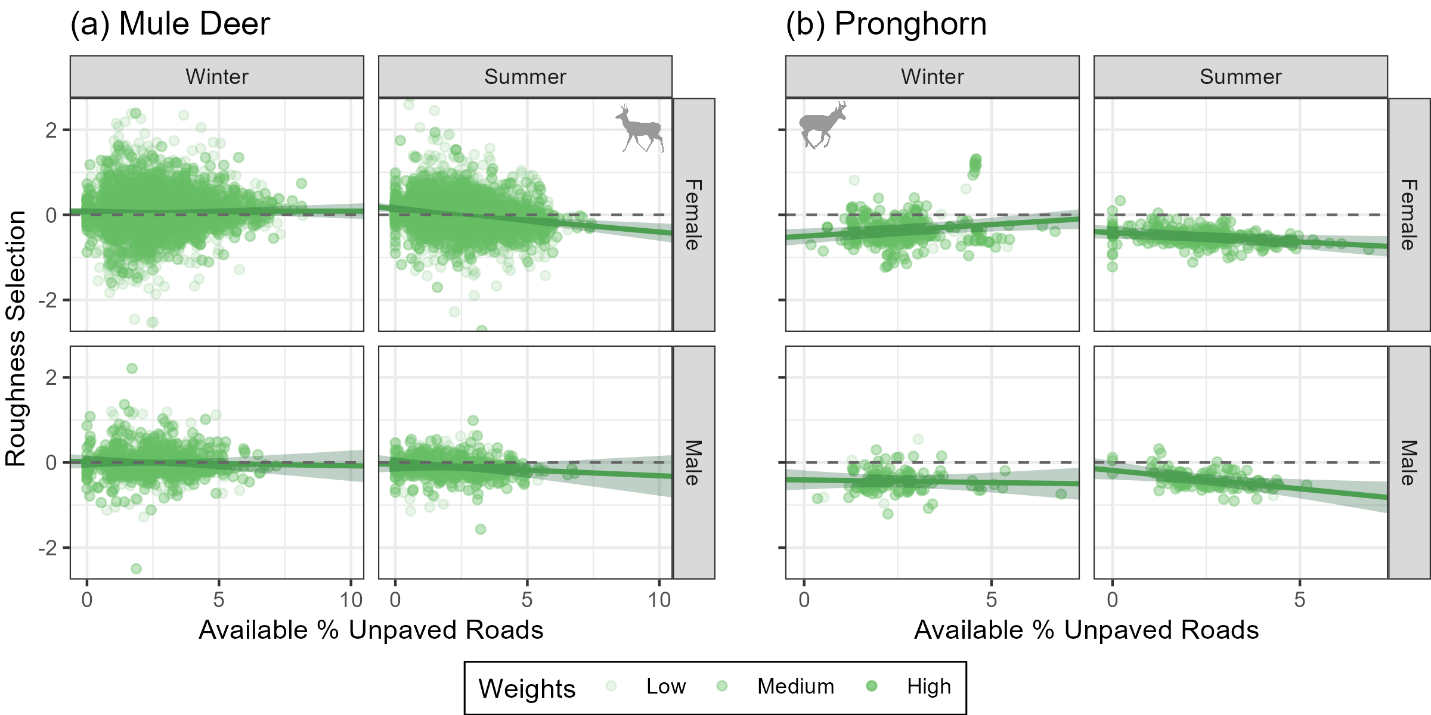


**Figure B10:** Mule deer (a) and pronghorn (b) selection for roughness in response to unpaved road density by sex (rows) and season (columns).

Like their female counterparts, male deer tended to select for forage, especially in the summer (winter: 0.004±0.05; summer: 0.264±0.12). These selection patterns were unaffected by both paved (male-winter: -0.021±0.04; male-summer: 0.036±0.10; Fig. B11a) and unpaved roads (female-winter: -0.006 ±0.02; male-winter: -0.015±0.04; female-summer: -0.090±0.05), with the exception of male deer in the summer, who increased their selection as unpaved road density increased (-0.091±0.11; Fig. B12a).

Male pronghorn, against expectations, avoided forage during both seasons, particularly in the summer (winter: -0.100±016; summer: -0.283±0.18). Increased paved road density did not significantly affect male pronghorn forage selection (winter: 0.024±0.20; summer: -0.036±0.32). Increased fence density, however, was significantly correlated with male pronghorn avoiding forage (winter: -0.316±0.20; summer: -0.169±0.22; Fig. B11b). Increased unpaved road density did not significantly affect pronghorn forage selection for either sex or season, apart from female pronghorn in winter who only slightly increased their avoidance of forage (female-winter: -0.161±0.09; male-winter: -0.082±0.15; female-summer: 0.015±0.11; male-summer: -0.118±0.21; Fig. B12b).


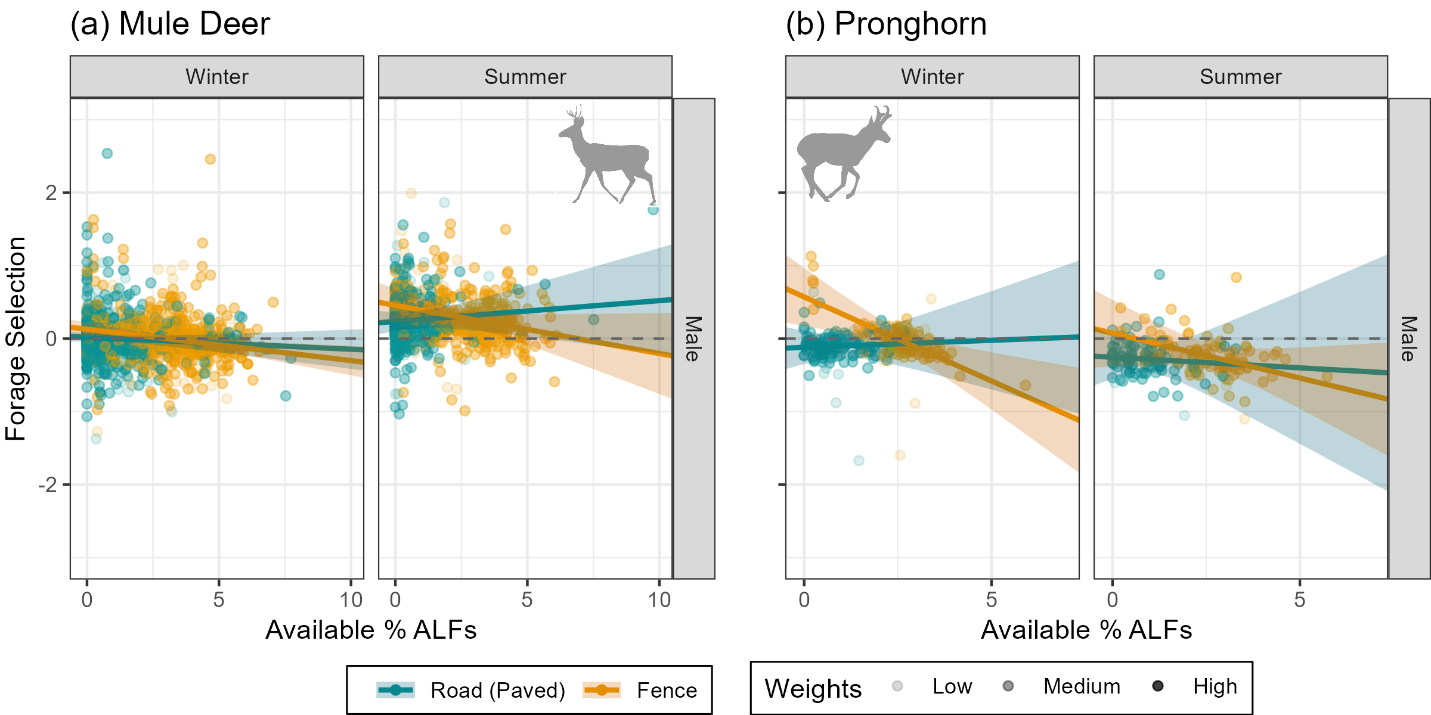


**Figure B11:** Male mule deer (a) and pronghorn (b) selection for forage in response to paved road (dark cyan) and fences (orange) density by season (columns).


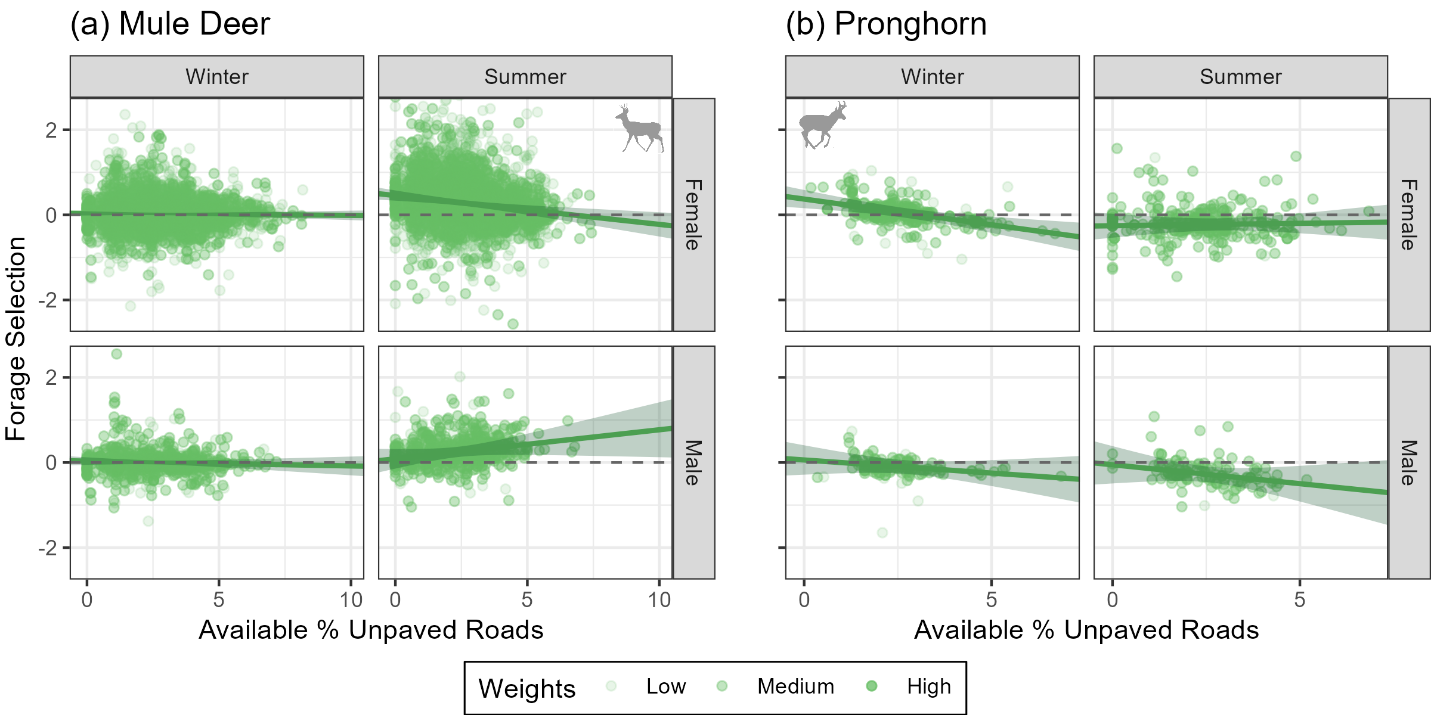


**Figure B12:** Mule deer (a) and pronghorn (b) selection for forage in response to unpaved road density by sex (rows) and season (columns).

Mule deer ten to avoid shrub cover in the winter (female: -0.117±0.03; male: -0.116±0.05) and select in the summer (female: 0.279±0.05; male: 0.067±0.10). Across sex and season, mule deer selection patterns for shrub cover did not significantly or intensely respond to increased paved road density (female-winter: -0.011±0.02; male-winter: -0.009±0.05; female-summer: -0.103±0.05; male-summer: -0.055±0.08), fence density (female-winter: -0.038±0.02; male-winter: -0.008±0.05; female-summer: 0.015±0.04; male-summer: 0.005±0.08), nor unpaved road density (female-winter: 0.034±0.02; male-winter: -0.025±0.05; female-summer: -0.024±0.04; male-summer: -0.022±0.09; Figs B13a, B14a).

Against expectations, pronghorn across sex and season strongly avoided shrub cover (female-winter: -0.087±0.06; male-winter: -0.178±0.09; female-summer: -0.429±0.06; male-summer: -0.319±0.09). In winter, this avoidance switched to selection as paved road density increased (female: 0.209±0.10; male: 0.212±0.15), though their selection remained unaffected by paved roads in the summer (female: 0.094±0.10; male: -0.015±0.15; Fig. B13b). As fence density increased, female pronghorn in the winter significantly increased their selection for shrub cover (0.174±0.08), switching from avoidance to selection. In all other seasons, however, pronghorn of either sex did not significantly change their shrub cover selection in response to fences (male-winter: -0.102±0.15; female-summer: -0.048±0.06; male-summer: -0.015±0.11; Fig. B13b). As unpaved road density increased, female pronghorn in winter significantly avoided shrub cover (-0.084±0.06) but increased unpaved road density was not significantly correlated with changes in shrub cover selection for the other seasons (male-winter: 0.050±0.11; female-summer: 0.017±0.06; male-summer: -0.031±0.10; Fig. B14b).


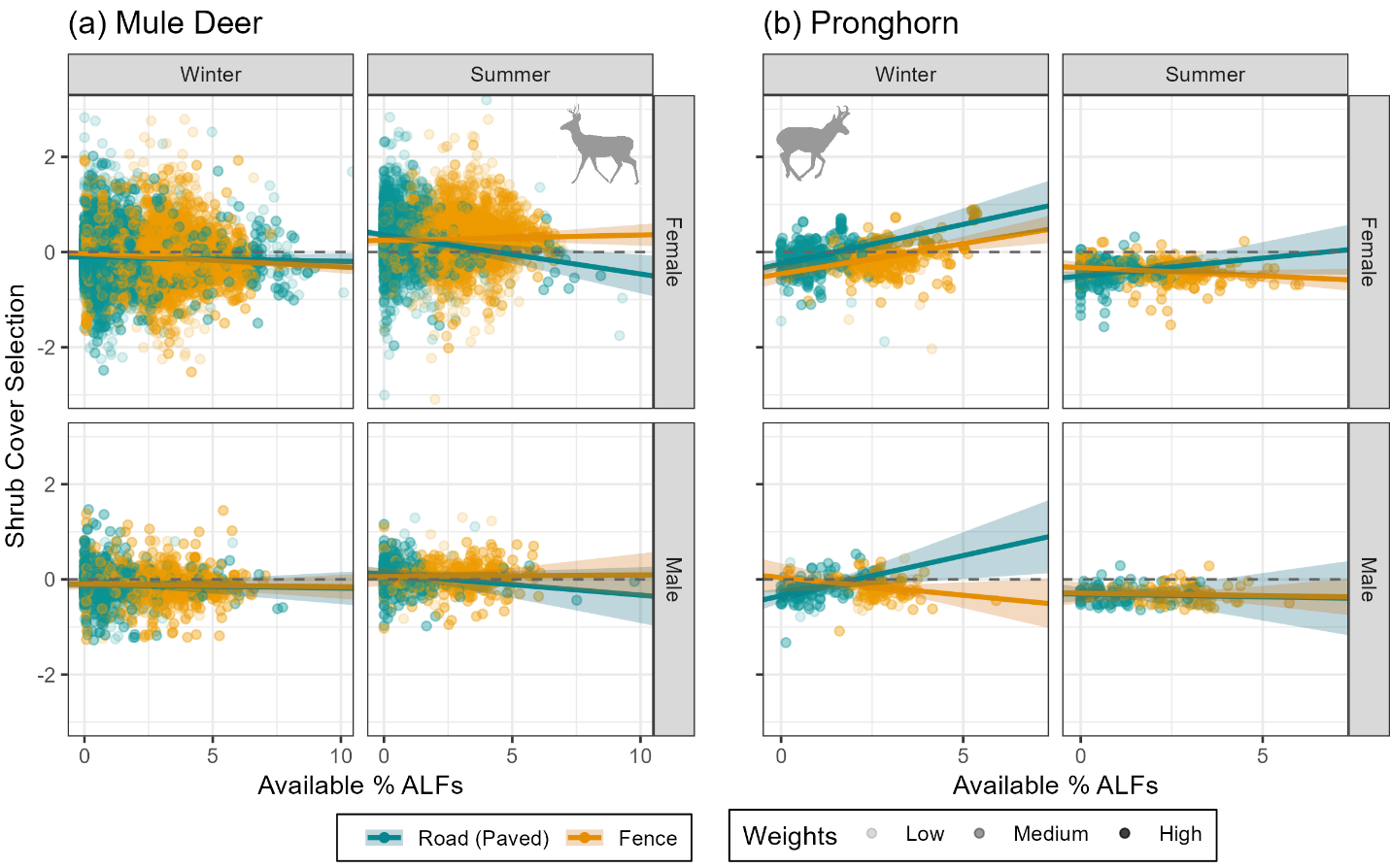


**Figure B13:** Mule deer (a) and pronghorn (b) selection for shrub cover in response to paved road (dark cyan) and fences (orange) density by sex (rows) and season (columns).


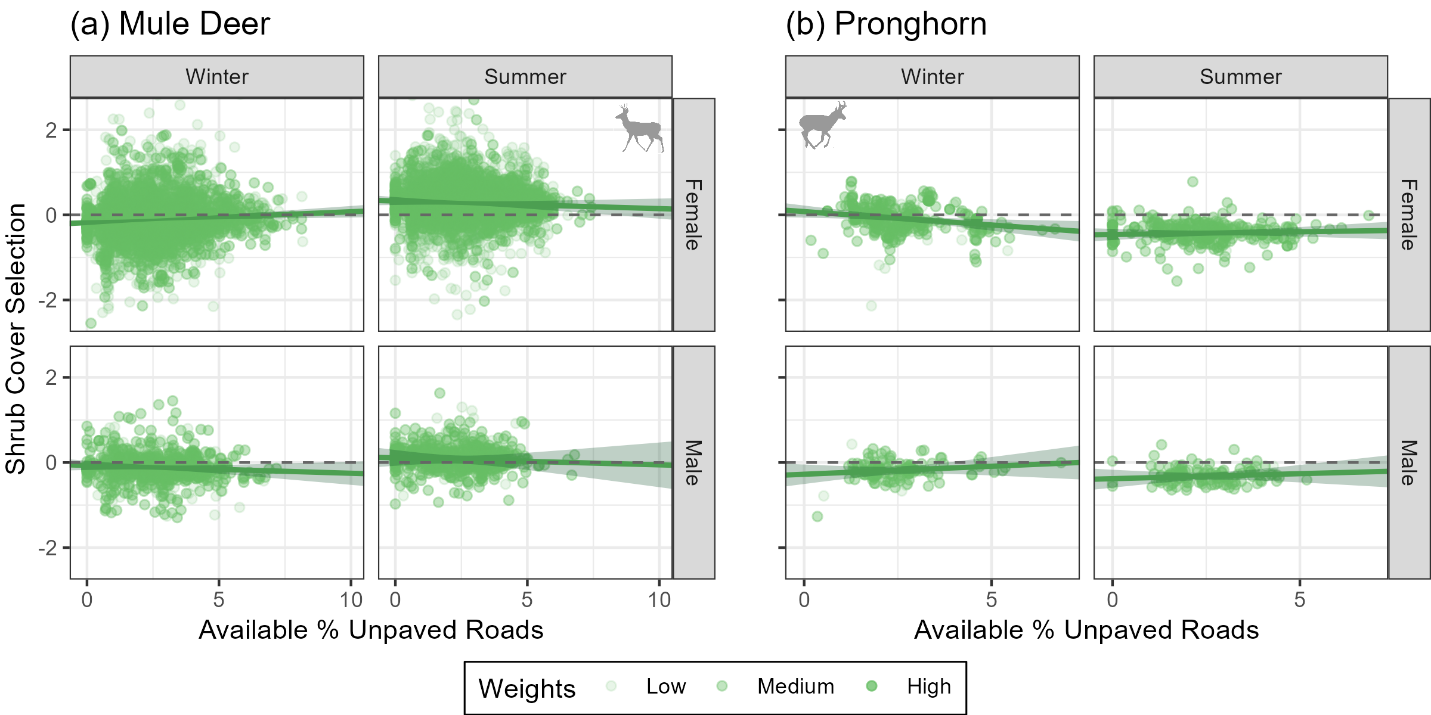


**Figure B14:** Mule deer (a) and pronghorn (b) selection for shrub cover in response to unpaved road density by sex (rows) and season (columns).

As expected, mule deer avoided tree cover in the winter and selected in the summer, however this was significant only for females (winter: -0.130±0.03; summer: 0.227±0.05) while male deer did not significantly select nor avoid trees (winter: 0.054±0.06; summer: 0.026±0.09). While female deer in the winter significantly increased their selection of tree cover as paved road density increased (0.045±0.02), this response was muted, and in all other sex-season combinations, deer tree selection did not respond significantly to paved road density (male-winter: 0.039±0.05; female-summer: -0.051±0.05; male-summer: 0.006±0.07). Fences similarly did not affect deer selection patterns for trees, apart from male deer in the summer who slightly increased their avoidance (female-winter: 0.020±0.03; male-winter: -0.006±0.05; female-summer: 0.007±0.04; male-summer: -0.084±0.08; Fig. B15a). Increasing unpaved road density, however, was significantly correlate with increased deer avoidance of tree cover, except for male deer in the summer whose selection patterns did not significantly change (female-winter: -0.041±0.03; male-winter: -0.060±0.05; female-summer: -0.096±0.04; male-summer: 0.075±0.08; Fig. B16a).

As expected, pronghorn significantly and intensely avoided tree cover across sex and season (female-winter: -1.264±0.05; male-winter: -1.238±0.05; female-summer: -1.133±0.06; male-summer: -1.025±0.07). Increased paved road density was significantly correlated with increased pronghorn selection for tree cover, apart from female pronghorn in the summer (0.037±0.05), however this response did not push their selection patterns from avoidance to selection (female-winter: 0.023±0.05; male-winter: 0.054±0.03; male-summer: 0.089±0.08). Increased fence density did not significantly affect pronghorn tree cover selection, except for females in the winter who significantly but not intensely increased their avoidance (female-winter: -0.040±0.02; male-winter: -0.004±0.03; female-summer: -0.013±0.03; male-summer: 0.014±0.05; Fig. B15b). Unpaved road density similarly did not impact pronghorn tree cover selection, with the exception of males in the summer who increased their selection, but this response did not push their selection patterns from avoidance to selection (female-winter: -0.007±0.01; male-winter: -0.022±0.02; female-summer: 0.017±0.03; male-summer: 0.063±0.05; Fig. B16b).


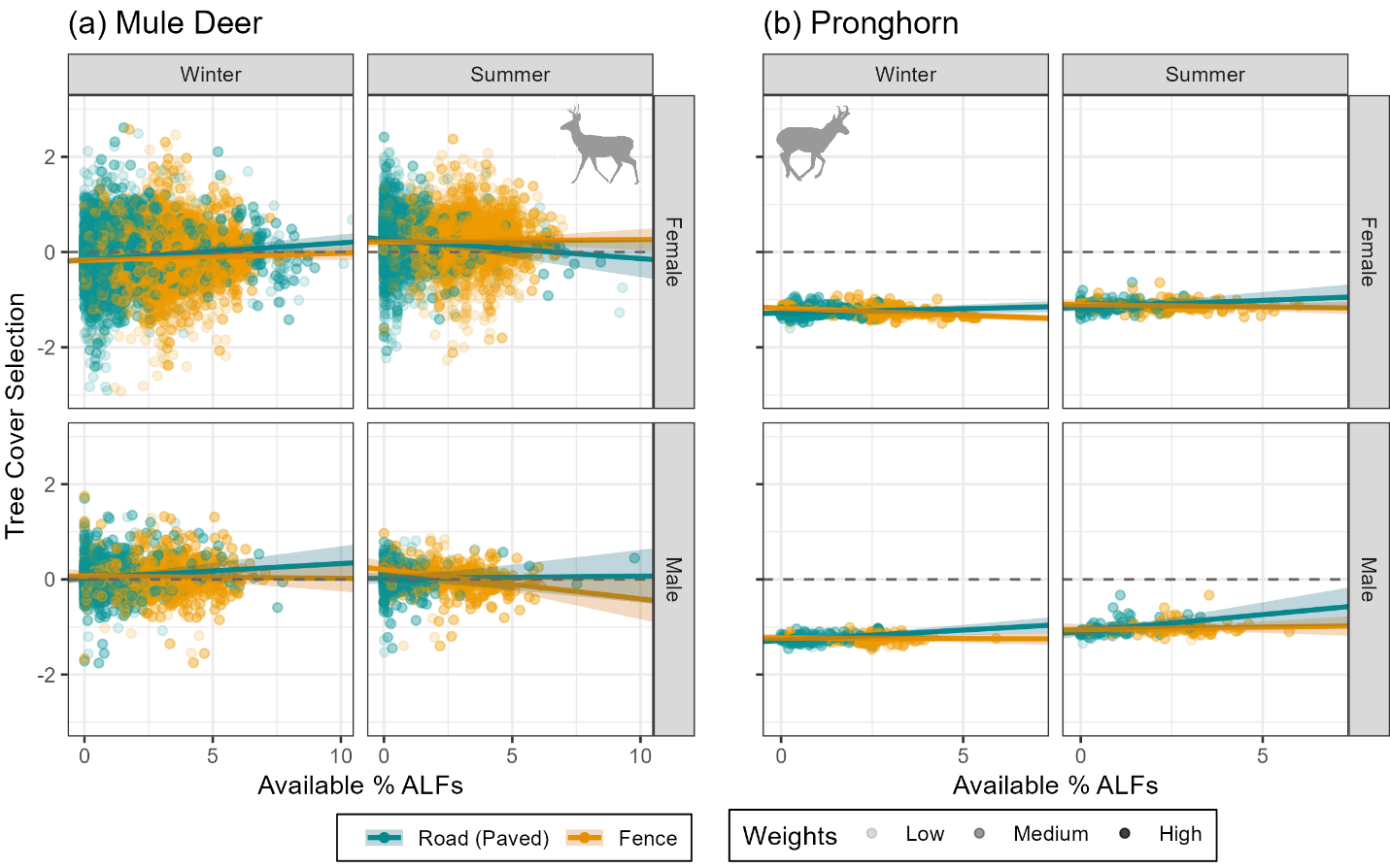


**Figure B15:** Mule deer (a) and pronghorn (b) selection for tree cover in response to paved road (dark cyan) and fences (orange) density by sex (rows) and season (columns).


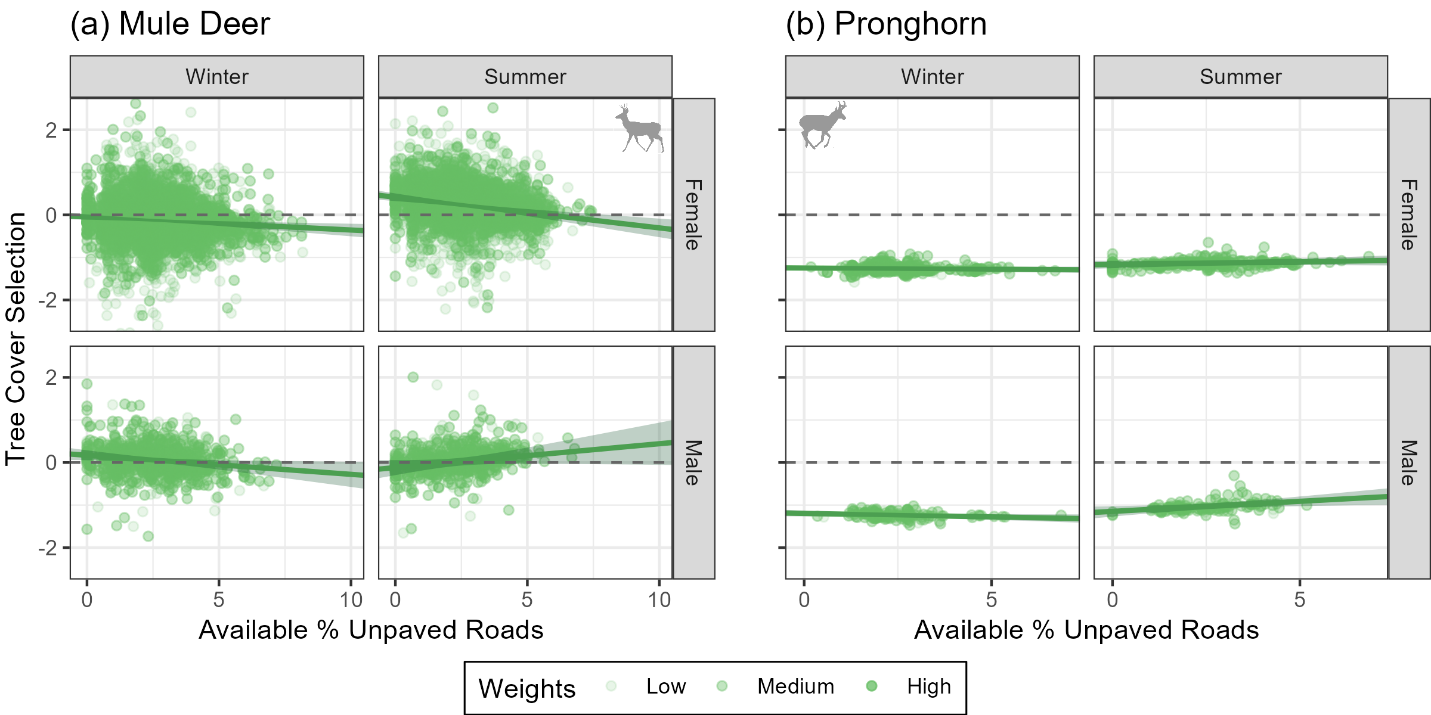


**Figure B16:** Mule deer (a) and pronghorn (b) selection for tree cover in response to unpaved road density by sex (rows) and season (columns).

Male deer did not show a particular selection nor avoidance of snow depth (0.050±0.08). Like their female counterparts, male deer significantly increased their avoidance of snow depth as paved road density increased (-0.118±0.07) and slightly increased their selection as fence density increased (0.089±0.07; Fig. B17a). Female mule deer significantly, but slightly, increased their selection of snow depth as unpaved road density increased (0.056±0.04) while male deer snow depth selection did not significantly respond to unpaved roads (-0.007±0.07).

Unlike their female counterparts, male pronghorn did not particularly select nor avoid snow depth (0.064±0.10). However, like the females, paved road density was significantly and intensely correlated with increased avoidance of snow depth (-0.573±0.15) and fence density was significantly correlated with increased selection (0.226±0.16; Fig. B17b). However, pronghorn of both sexes did not significantly change their snow depth selection patterns as unpaved road density increased (female: -0.018±0.07; male: -0.028±0.12; Fig. B18b).


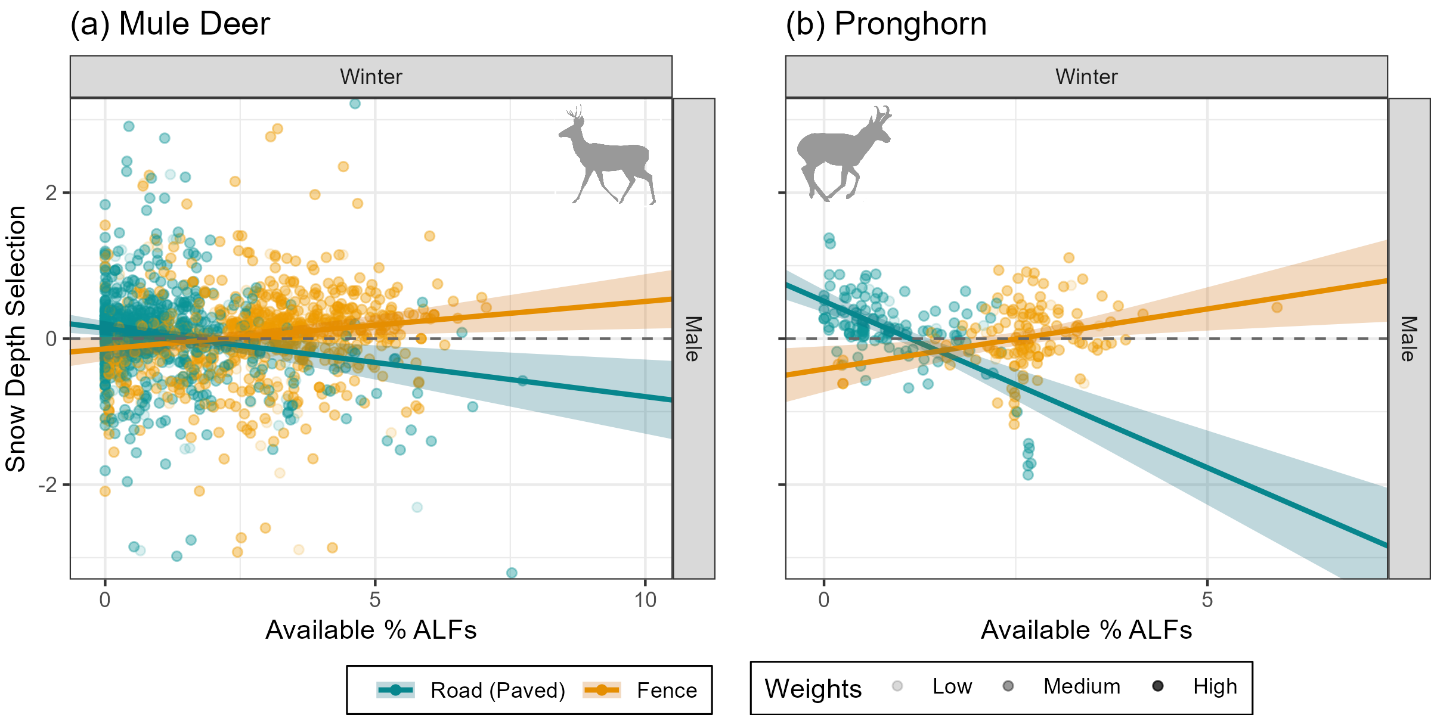


**Figure B17:** Male mule deer (a) and pronghorn (b) selection for snow depth in response to paved road (dark cyan) and fences (orange) density.


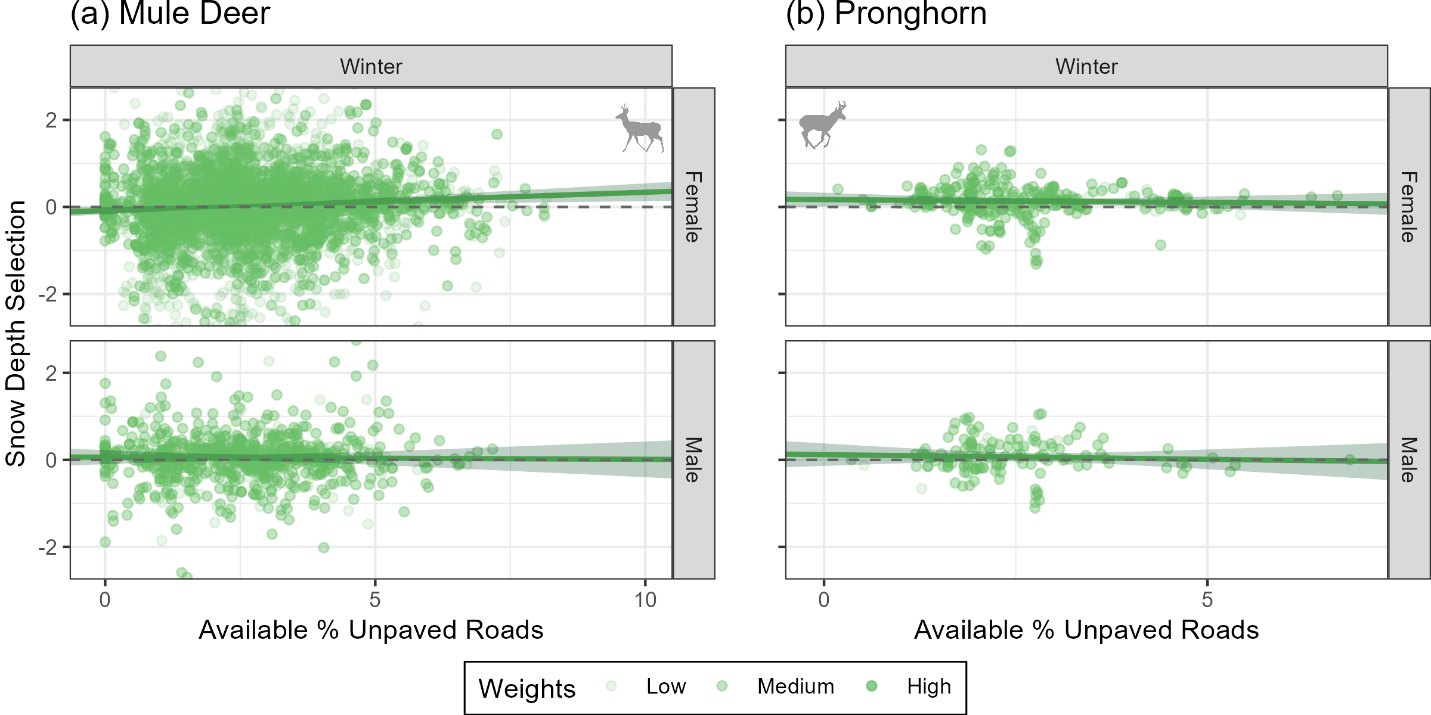


**Figure B18:** Mule deer (a) and pronghorn (b) selection for snow depth in response to unpaved road density by sex (rows).

# Appendix C: Utah environment

## C.1 General Utah climate and ecology

The state of Utah, U.S.A. is home to a wide diversity of terrain, climate, and ecology. Its mountains and plateaus extend north-to-south across most of the state, and its highest ranges, the Uinta Mountains, run east to west in the northeast corner of the state. Desert mesas and canyons and found in most of the southern and eastern regions of Utah and desert valleys cover most of the western portion of the state [1].

The environments in Utah are generally driven by climate, or more specifically, precipitation and temperature (Figs, C1, 2), which in turn are typically dictated by elevation [1, 2]. Utah is made up of seven climatic divisions, as defined by the National Climate Data Center (NCDC): Western, Dixie, North Central, South Central, Northern Mountains, Uinta Basin, and Southeast (Fig. C3). Most of Utah (particularly the Western, Dixie, Southeast, and Uinta Basin) is defined as semiarid steppe, meaning that summers are hot and dry and winters are cold with average annual precipitation ranging from 12.5 to 38 cm (5 to 15 in) [1]. The higher elevational valleys and mountains of the state (particularly the Northern Mountains) are characterized by warm-to-hot summers, very cold winters with large amounts of snowfall, and typically no dry season, with average annual precipitation ranging from 25.5 to 140 cm (10 to 55 in) [1]. For most of the climatic regions of Utah, the winter seasons bring in the most precipitation, typically in the form of snowfall, and the summer seasons have drier weather [1].

Utah is home to seven level III ecoregions: Central Basin and Range, Mojave Basin and Range, Wyoming Basin, Wasatch and Uinta Mountains, Colorado Plateaus, Southern Rockies, and Northern Basin and Range. These seven level III ecoregions can be broken up further into 37 level IV ecoregions (Fig. C3) [3]. These ecoregions describe the wide ecological diversity of the deserts, canyonlands, salt flats, wetlands, shrublands, valleys, woodlands, and alpine areas of Utah.

The Central Basin and Range ecoregion makes up the majority of western Utah and comprises the valleys, deserts, shrublands, marshes, and wetlands around, west, and south of the Great Salt Lake and Utah Lake, the woodland and shrubland foothills west of the Wasatch Front, and a large majority of the populated areas of Utah, notably Salt Lake City. In the valleys, the dominant vegetation are shrubs such as saltbush-greasewood (*Sarcobatus spp.)* and basin big sagebrush (*Artemisia tridentata ssp. tridentata)*, while higher elevation forests are composed of pinyon-juniper and western spruce-fir woodlands. Much of the land in this region is used for livestock grazing.

The basins and mountains of the Mojave Basin and Range are found in the southwest corner of Utah and are generally lower in elevation, warmer and drier than those of the Central Basin and Range. The dominant vegetation in this region’s basins is creosote bush (*Larrea tridentata*) and pinyon-juniper woodlands are found in higher elevations. Most of this region is federally-owned and managed.

The Wyoming Basin is found in the northeast corners of Utah and are comprised of grasslands and shrublands within rolling plains, hills, mesas, sagebrush steppe, and low mountains. Unlike many of the other higher-elevation ecoregions in Utah, the Wyoming Basin lacks extensive forests and woodlands, and thus much of this region is used for livestock grazing and oil and natural gas fields.

The Wasatch and Uinta Mountains is a high-elevation montane ecoregion that runs north to south through the central regions of Utah and includes the glaciated Uinta Mountains, the Wasatch Range, and the Wasatch Plateau. These ranges above 11,000 feet, particularly in the Uintas, are composed of alpine meadows, rockland, and talus fields. Between 8,000 and 11,000 feet, these mountains are dominated by forests composed of Douglas-fir (*Pseudotsuga menziesii*), quaking aspen (*Populus tremuloides*), Engelmann spruce (*Picea engelmannii*), and subalpie fir (*Abies lasiocarpa*) with mixes of ponderosa pine (*Pinus ponderosa*) and limber pine (*Pinus flexilis*). Between 5,000 and 8,000 feet, forests are comprised mainly of pinyon-juniper and mahogany-oak scrub communities. This ecoregion is used for logging, summer grazing, and recreation.

The Colorado Plateaus make up the majority of eastern Utah and is geologically composed of benches, mesas, buttes, salt valleys, cliffs, and canyons with thick layers of sedimentary rock. Higher elevations are dominated by pinyon-juniper woodlands and lower elevations are composed of saltbush-greasewood and blackbrush (*Coleogyne ramosissima*) communities. Summer thunderstorms help support a high diversity of grasses and other endemic plants that are not found in the Central Basin and Range. Many of Utah’s national parks are found in this ecoregion and bring in thousands to millions of visitors annually.

The Southern Rockies in Utah are isolated islands of mountains found within the Colorado Plateaus in the southeast of Utah. Lower to middle elevations are used as livestock grazing and are composed of Gambel oak (*Quercus gambelii*), ponderosa pine, and mountain sagebrush (*A. tridentata ssp. vaseyana*). Higher elevations are dominated by subalpine fir, Engelmann spruce, Douglas-fir, aspen, and mountain brush and are not as heavily grazed.

The Northern Basin and Range is found in the northwest corner of Utah and is geologically composed of lava plains, rolling hills, alluvial fans, valleys, and some mountains. Lower elevations are dominated by sagebrush steppe vegetation, and the mountains are composed of mountain sagebrush, Idaho fescue (*Festuca idahoensis*), Douglas-fir, subalpine fir, aspen, and juniper (*Juniperus spp.*).

About 50% of all mule deer summer home ranges were found in the Wasatch and Uinta Mountains ecoregion (most of which were in the Semiarid Foothills, High Plateaus, and Wasatch Montane Zone sub-ecoregions), followed by 21% in the Central Basin and Range (most of which were in the Woodland and Shrub-Covered Low Mountains, Sagebrush Basins and Slopes, and High Elevation Carbonate Mountains sub-ecoregions) and 19% in the Colorado Plateaus (most of which were in the Semiarid Benchlands and Canyonlands, Escarpments, and Northern Uinta Basin Slopes sub-ecoregions). About 39% of all mule deer winter home ranges were found in the Colorado Plateaus (the same sub-ecoregions as the summer ranges in the Colorado Plateaus were in), with 27% of the other winter ranges in the Central Basin and Range (with the same sub-ecoregions as the summer ranges, with the exception of Shadscale-Dominated Saline Basins). 40% and 45% of all pronghorn summer and winter home ranges, respectively, were found in the Central Basin and Range (specifically the Sagebrush Basins and Slopes, Shadscale-Dominated Saline Basins, and Woodland- and Shrub-Covered Low Mountains). 31% and 34% of all pronghorn summer and winter ranges, respectively, were then found in the Wasatch and Uinta Mountains (specifically the High Plateaus, Semiarid Foothills, and Mountain Valleys), and 19% and 15% of remaining pronghorn summer and winter ranges, respectively, were found in the Wyoming Basin (specifically the Foothill Shrublands and Low Mountains, Sub-Irrigated High Valleys, and Rolling Sagebrush Steppe sub-ecoregions).

## C.2 Usual Utah snow depth in comparison to snow depth during the study period

We sought to understand if snow depths during the study period (2013-2021) were different than usual snow depths in previous years. We first downloaded data from SNODAS, the dataset we used in the main text [5], for each day in February for all years that SNODAS was available (2004) to the end of the study period (2021). For some years, snow depth was not available and only snow water equivalent (SWE) was available, and so we downloaded both snow depth and SWE for this time period. This resulted in 190 layers of snow depth and 212 layers of SWE. We then calculated the mean and standard deviation for both snow depth and SWE (Fig. C5).

Because the different areas of Utah have such a wide range of annual precipitation (Fig. C2) and thus snow depths, we wanted to compare by the area a pronghorn or mule deer would see and use rather than across the whole state. To do so, we clipped each year’s February monthly snow depth (see section 2.4 in the main text) and SWE to the availability domain of each winter home ranges (see section 2.6 in the main text) of that year and calculated the mean Z-score, or the number of standard deviations by which the snow of that year’s availability domain differed from the mean [6], within the availability domain. This z-score would thus reflect whether the area that is available to the individual mule deer or pronghorn differed significantly from the mean snow depth or SWE of previous years in that same area. We then calculated the median z-score for each year and plotted each year’s z-score as boxplots to see if certain years varied significantly from the mean or not.

The median z-score for all home range’s availability domains in 2013 was 3.29, for 2014 it was -0.41, for 2015 it was -0.70, for 2016 it was 0.33, for 2017 it was -0.21, for 2018 it was -0.45, for 2019 it was 0.72, for 2020 it was -0.31, and for 2021 it was -0.39 (Fig. C6). 2013 was the wettest year of this study period and 2015 was the driest year. With the exception of 2013, most of the years of our study period were within one standard deviation of the mean, meaning that the precipitation as snow during our study period was not significantly different than normal.

**References**

1. Gillies, R.R., and R. D. Ramsey. 2009. Climate of Utah. In Rangeland Resources of Utah, 2nd ed., R.E. Banner, B.D. Baldwin, and E.I.L. McGinty editors.

2. Ramsey, R. D. and N.E. West. 2009. Vegetation of Utah. In Rangeland Resources of Utah, 2nd ed., R.E. Banner, B.D. Baldwin, and E.I.L. McGinty editors.

3. Woods, A.J., Lammers, D.A., Bryce, S.A., Omernik, J.M., Denton, R.L., Domeier, M., and Comstock, J.A., 2001, Ecoregions of Utah (color poster with map, descriptive text, summary tables, and photographs): Reston, Virginia, U.S. Geological Survey (map scale 1:1,175,000).

4. Abatzoglou, J.T., S.Z. Dobrowski, S.A. Parks, K.C. Hegewisch, 2018, Terraclimate, a high-resolution global dataset of monthly climate and climatic water balance from 1958-2015, Scientific Data 5:170191, [doi:10.1038/sdata.2017.191](https://doi.org/10.1038/sdata.2017.191)

5. National Operational Hydrologic Remote Sensing Center. Snow data assimilation system (SNODAS) data products at NSIDC, version 1. 2004; Available from: <https://nsidc.org/data/G02158/versions/1>

6. Abdi, H. (2007). Z-scores. *Encyclopedia of measurement and statistics*, *3*, 1055-1058.


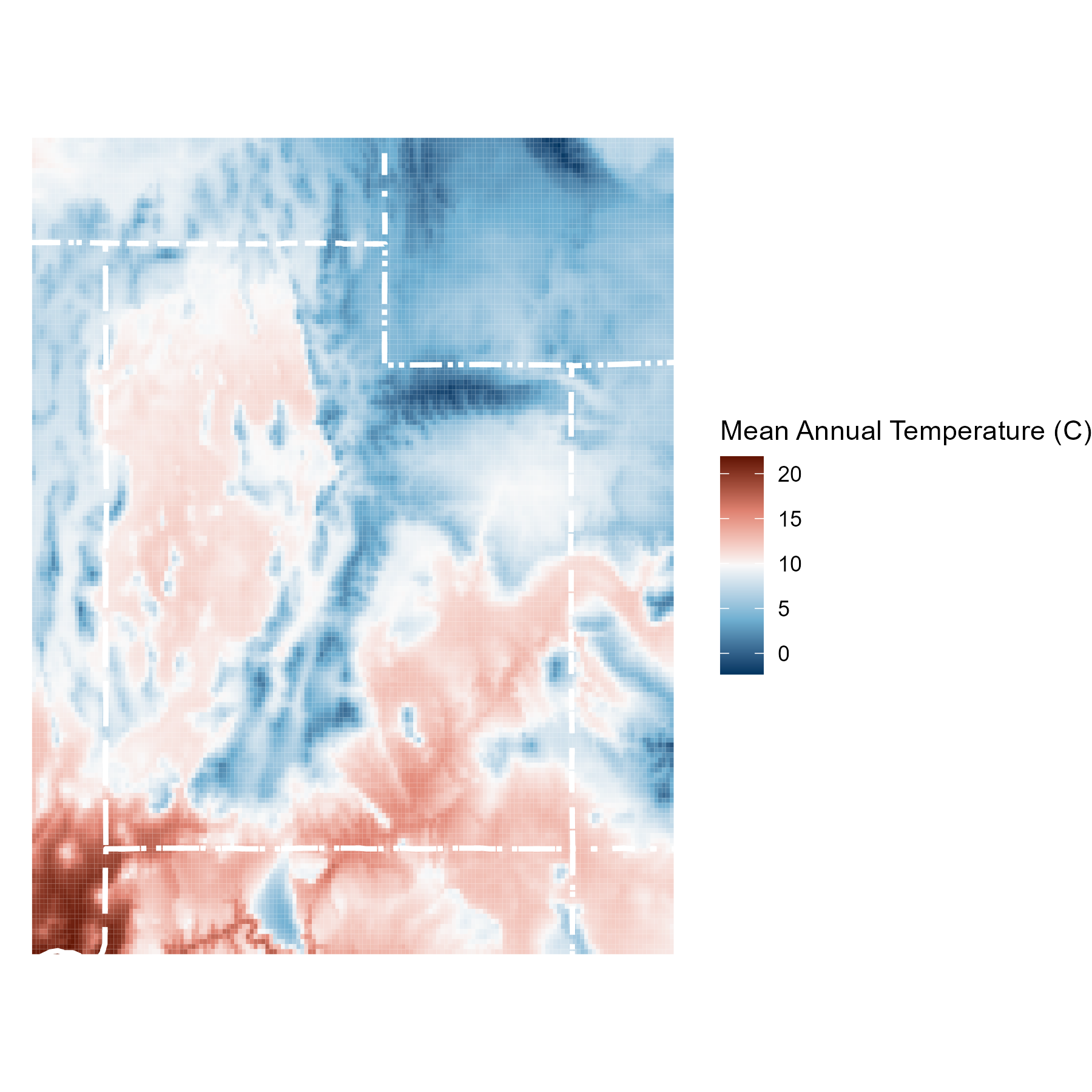


**Figure C1:** Mean annual temperature (°C) of Utah and surrounding states. State borders are delineated with a dotted white line. Data was acquired from TerraClimate [4].


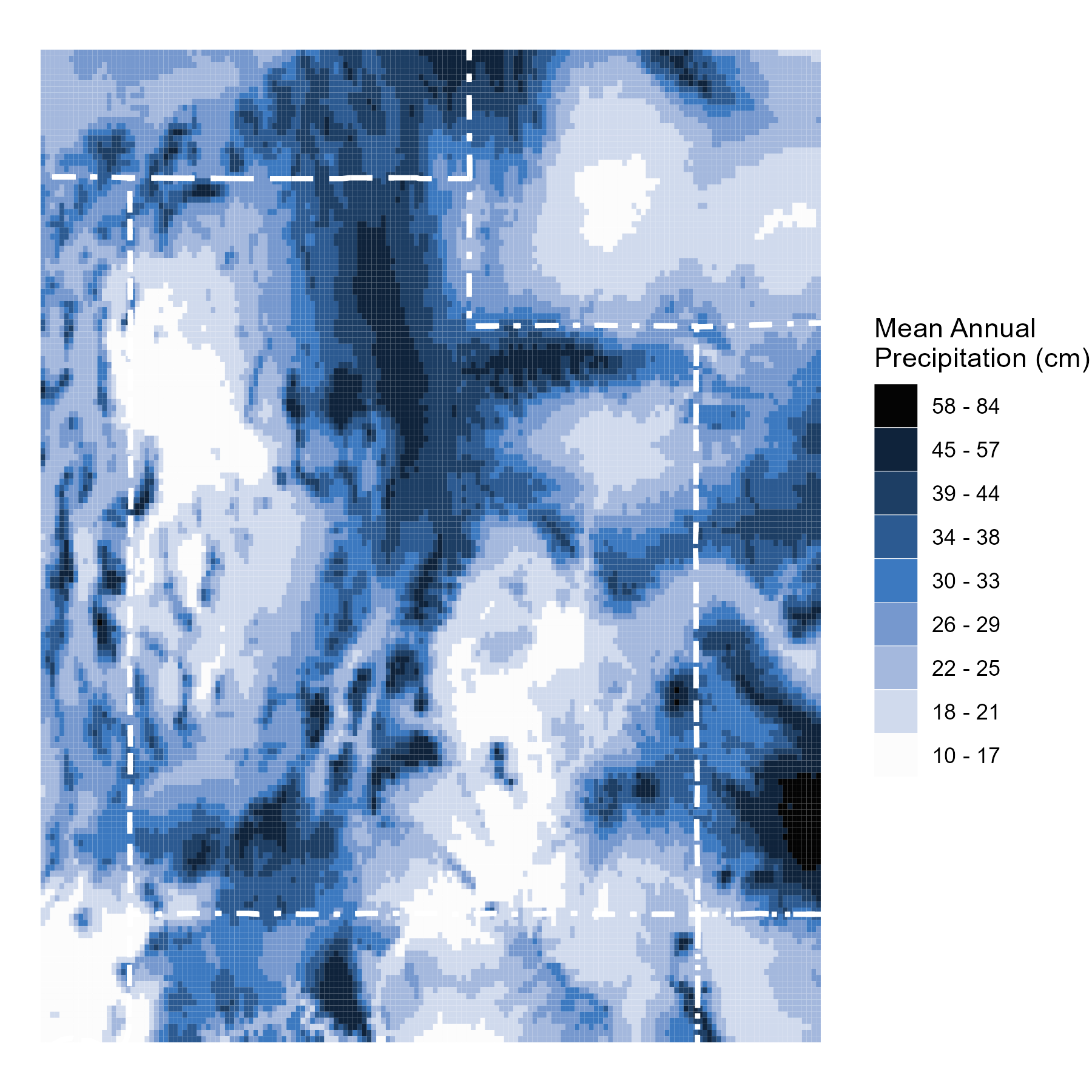


**Figure C2:** Mean annual precipitation (cm) of Utah and surrounding states. State borders are delineated with a dotted white line. Data was acquired from TerraClimate [4].


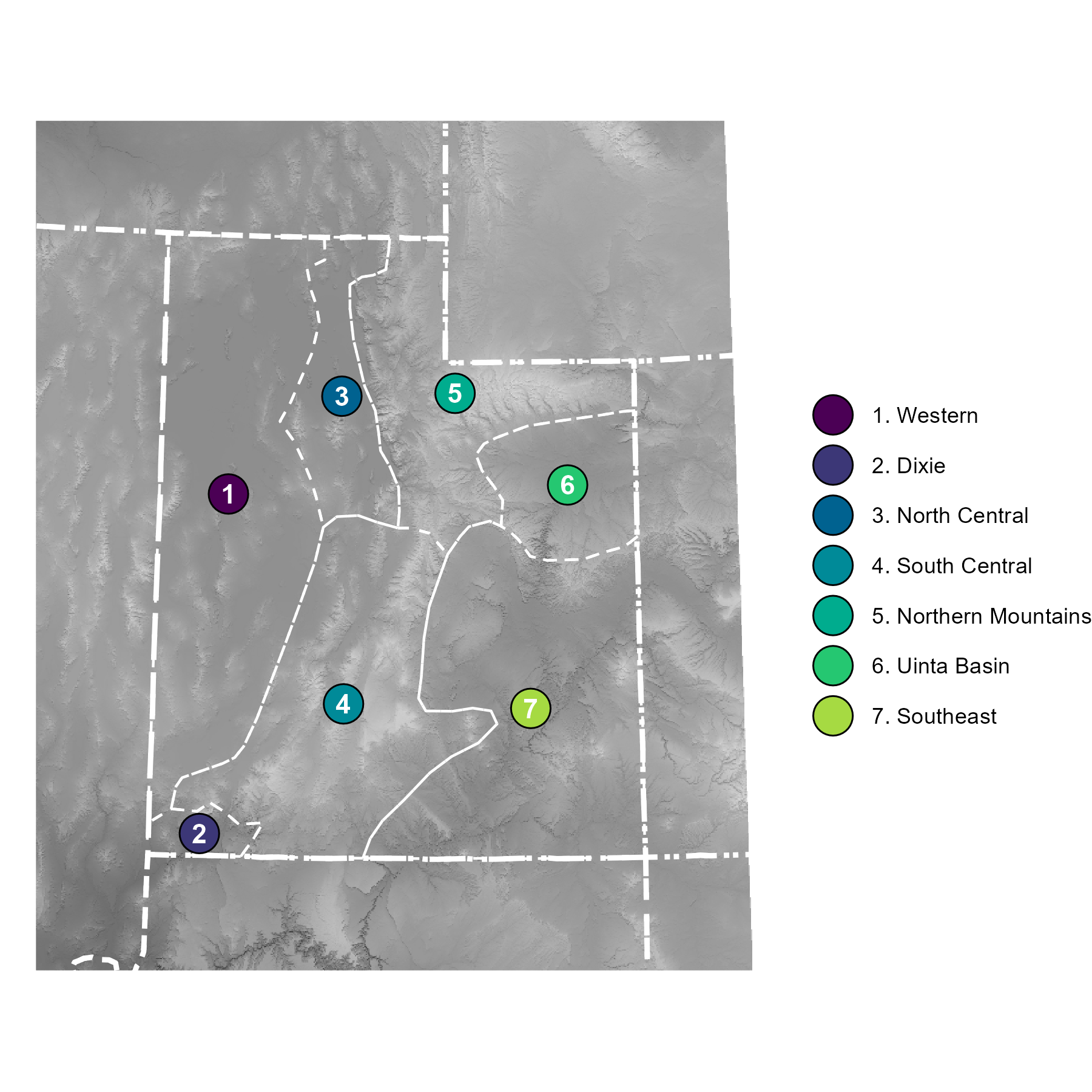


**Figure C3**: Climate regions of Utah, with elevation basemap. Thick lines denote state boundaries while thin lines denote climate regions.


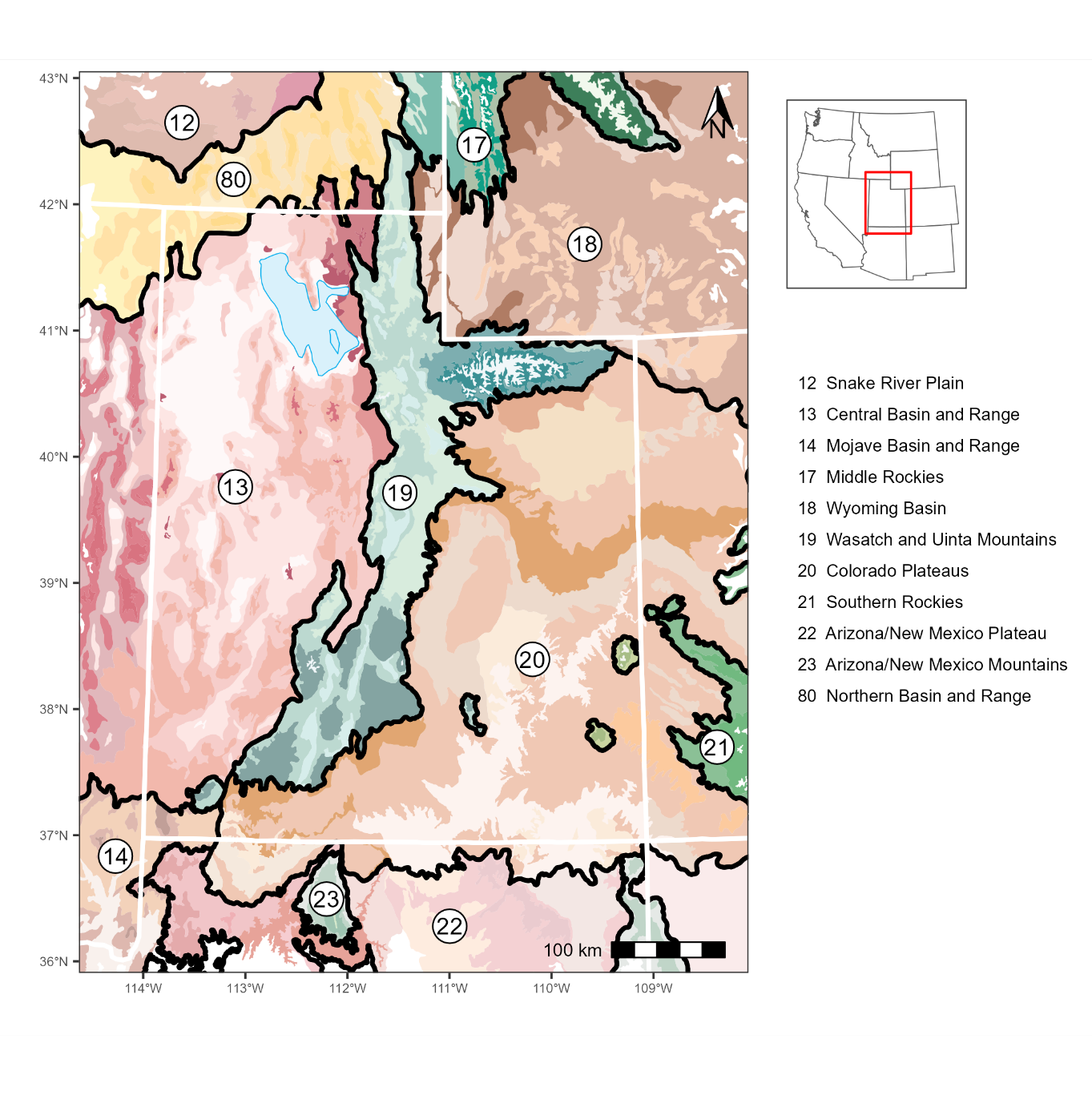


**Figure C4:** Ecoregion map of Utah, USA and surrounding states. The white boundaries delineate the states, the dark boundaries and corresponding numbers delineate and indicate to the Level III ecoregions, and the colors within the boundaries indicate the Level IV ecoregions.


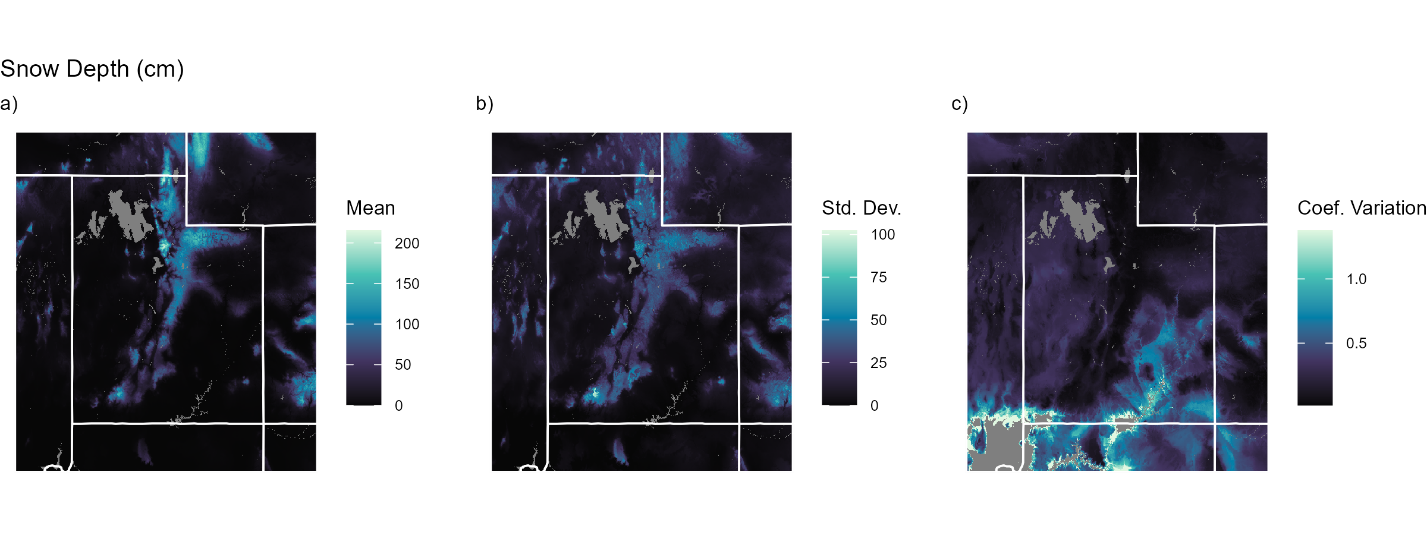

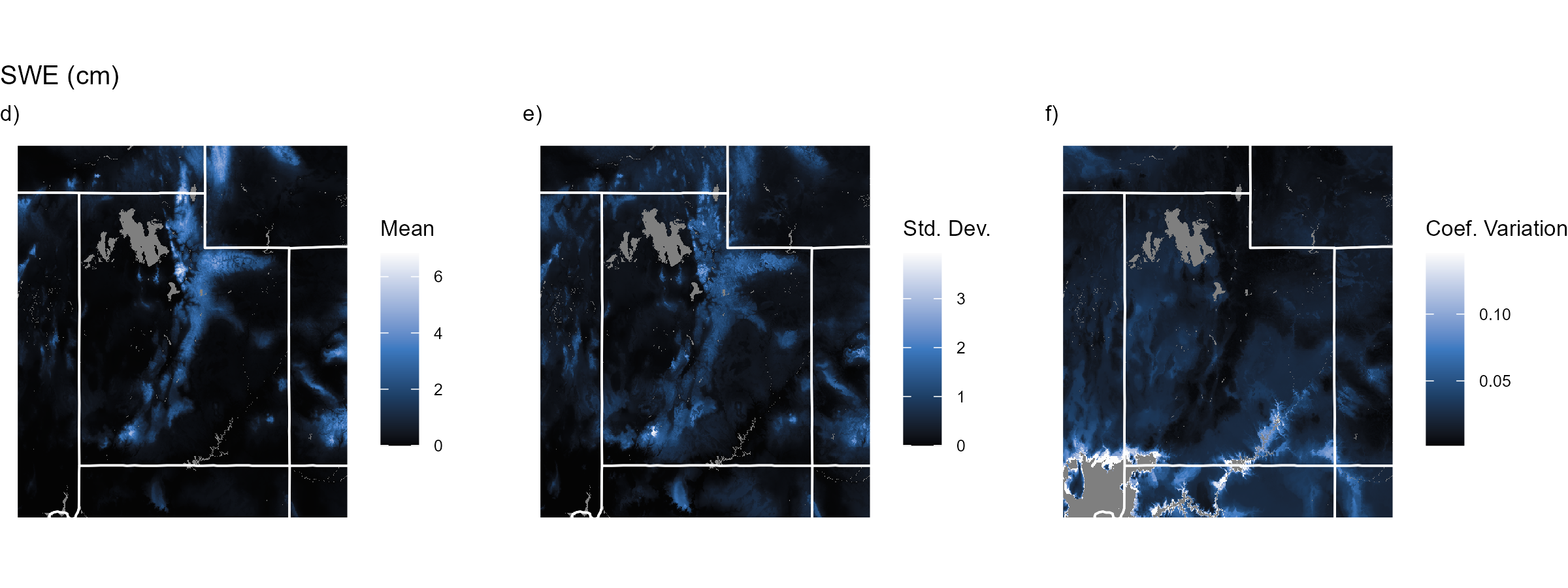


**Figure C5:** Mean (a,d), standard deviation (b,e), and coefficient of variation (c,f) of snow depth (top row) and snow water equivalent (SWE) from 2004-2021 in Utah and surrounding states (state boundaries delineated by white lines). Data from SNODAS [5].


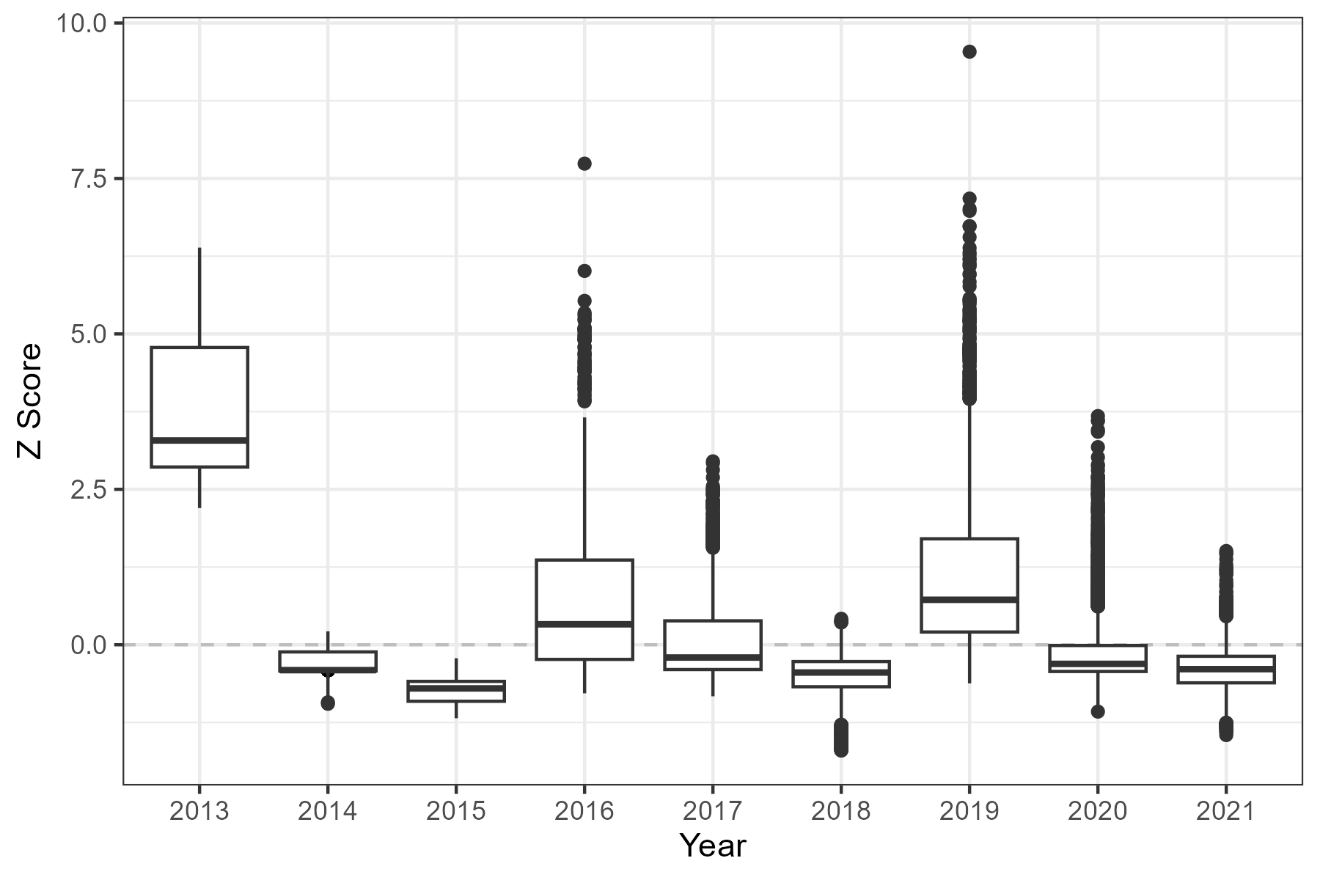


**Figure C6:** Boxplots of z-scores of the snow depth and snow water equivalent (SWE) in the availability domains of each year’s home ranges for pronghorn and mule deer. A z-score reflects how many standard deviations a value is from the mean.
